# Influenza A Virus H5N1 Subtype: Resurgent Interspecies and Intercontinental Transmission, and a New Host

**DOI:** 10.3390/pathogens15010006

**Published:** 2025-12-20

**Authors:** Matloob Husain

**Affiliations:** Department of Microbiology and Immunology, University of Otago, Post Box 56, Dunedin 9054, New Zealand; matloob.husain@otago.ac.nz

**Keywords:** influenza A virus, H5N1 subtype, clade 2.3.4.4b, interspecies, intercontinental, transmission, avian, migratory birds, cow, cow milk

## Abstract

It has been more than 25 years since the avian influenza A virus (IAV) H5N1 subtype emerged in humans in 1997. Since then, this virus has become endemic in poultry and wild birds and has been causing sporadic infections in humans. Furthermore, the H5N1 subtype has undergone numerous reassortment events with other avian IAVs, resulting in the emergence of various H5Nx subtypes. Furthermore, the original H5 hemagglutinin (HA) has evolved genetically and antigenically and diversified into multiple lineages, phylogenetic clades, and subclades. In 2020, clade 2.3.4.4b H5N1 emerged in Europe and spread intercontinentally. Lately, H5N1 has exhibited a resurgence in transmission across the continents in different avian and mammalian species. Importantly, to the surprise of influenza virologists, H5N1 has recently been found to infect a new host, the cow, and has been detected in cow milk. Furthermore, spillover infections of H5N1 have also been detected in dairy farm workers. This review summarizes the recent transmission of clade 2.3.4.4b H5N1 across the globe and its pathogenesis and adaptation in different hosts. Also, this review discusses the susceptibility of the H5N1 subtype to anti-IAV drugs and vaccines and the public health response and measures that are undertaken and can be taken in the future to contain its further spread.

## 1. Introduction

The influenza virus has been a globally prevalent human and animal pathogen for centuries and has the potential to remain so for centuries to come [1]. The influenza virus transmits through aerosol and causes recurring seasonal epidemics and occasional pandemics of influenza, or flu, an acute febrile upper respiratory tract disease in humans. The seasonal flu epidemics cause more than one billion cases, 9 million hospitalizations, and an average of 389,000 deaths each year worldwide [2]. In addition, these epidemics impose a significant economic burden on the global economy because of the direct medical costs and loss of productivity due to absenteeism [2]. In the event of a flu pandemic, these numbers and burden increase manyfold. Furthermore, the health and economic burden of flu is more severe in young children and elderly people and people living with underlying medical conditions, e.g., chronic pulmonary diseases, and in low socio-economic status communities [2]. Occasionally, the avian and swine influenza viruses also cross the ecological barrier and cause spillover zoonotic infections in humans through close contact with infected animals [2]. While the zoonotic infections in humans by swine influenza viruses are generally abortive, such infections by avian influenza viruses can be deadly, with a mortality rate up to 50% [2].

The influenza virus is an enveloped virus with a linear but segmented negative-sense, single-stranded RNA genome and belongs to the *Orthomyxoviridae* family of viruses [1]. Influenza virus exists in four types—A, B, C, and D—based on the antigenic characteristics of their internal antigen, nucleoprotein (NP) [2,3]. The influenza A virus (IAV) has a broad host range, and, in addition to humans, it infects pigs, dogs, cats, horses, bats, and marine mammals, and different species of birds [2]. Therefore, IAV hosts inhabit all three media of the environment—land, air, and water. Consequently, IAV constantly circulates in nature and undergoes regular interspecies transmission, causing all three flu events—epidemics, pandemics, and zoonotic outbreaks [2]. The influenza B and C viruses mainly infect humans, whereas the influenza D virus mainly infects cattle, small ruminants, and pigs. Influenza B virus also causes seasonal epidemics, but influenza C and D viruses have not been associated with recurring seasonal epidemics. Similarly, influenza B, C, and D viruses have not been associated with pandemics. So far, IAV has been documented to cause four pandemics—in 1918, 1957, 1968, and 2009 [2]—and remains a pandemic threat. Therefore, IAV is the most researched influenza virus, and the rest of the information below is derived from the IAV literature.

**Figure 1:**
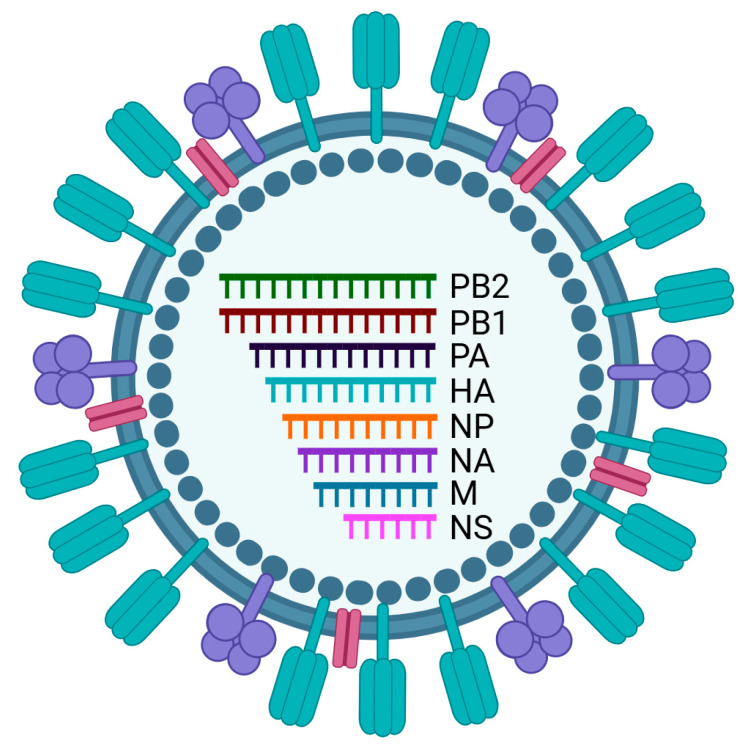
Diagram showing the composition of an influenza A virus (IAV) particle. The enveloped IAV particle contains the main surface antigens hemagglutinin (HA), shown in turquoise; neuraminidase (NA), shown in purple; and ion channel M2, shown in magenta, inserted in the envelope (shown in gray). Underneath the envelope, M1 forms a continuous layer, shown in gray, dots. In the center, eight vRNPs, shown in different colors and named, are located. Illustration created with BioRender.com.

IAV particles are generally spherical but can be filamentous and contain two surface antigens, hemagglutinin (HA) and neuraminidase (NA) membrane glycoproteins in the envelope (Figure 1). The IAV envelope also contains another membrane protein, matrix protein 2 (M2), which forms an ion channel (Figure 1). Underneath the envelope, matrix protein 1 (M1) forms a continuous layer (Figure 1), and at the center of the particle, eight viral ribonucleoprotein (vRNP) complexes are located (Figure 1). Each vRNP is composed of an RNA gene segment, namely polymerase basic 2 (PB2), polymerase basic 1 (PB1), polymerase acidic (PA), HA, NP, NA, matrix (M), or non-structural (NS), and NP protein, and three RNA polymerase proteins—PB2, PB1, and PA [1]. To infect the host cells, IAV attaches to the host cell surface by binding to α2,3-linked or α2,6-linked sialic acid receptors (in humans, pigs, and birds) or MHC class II receptors (in bats) through the HA receptor-binding protein and then enters the cell by endocytosis [4]. IAV replicates its RNA genome in the host cell nucleus and subsequently assembles its progeny on the host cell plasma membrane and releases them by budding [1].

## 2. IAV Subtypes

IAV is further subtyped based on the antigenic properties of HA and NA, and those IAV subtypes are known as, e.g., H1N1, H2N1, and H3N2 [3]. So far, 18 HA and 11 NA subtypes have been identified and can be divided into different phylogenetic groups [2]. For HA, the H1, H2, H5, H6, H8, H9, H11, H12, H13, H16, H17, and H18 subtypes have been placed in phylogenetic group 1, whereas the H3, H4, H7, H10, H14, and H15 subtypes have been placed in phylogenetic group 2. Similarly, for NA, the N1, N4, N5, and N8 subtypes are in placed phylogenetic group 1, the N2, N3, N6, N7, and N9 subtypes in phylogenetic group 2, and the N10 and N11 in phylogenetic group 3 [2].

The IAVs with HA subtypes, H1–H16, and NA subtypes, NA1-NA9, have been isolated from various species of wild birds, of which the aquatic birds are the main reservoir host of these viruses [2]. Also, avian IAV subtypes H5–H7, H9, H10, N1–N4, and N6–N9 have been isolated from humans. However, only H1N1 (in 1918 and 2009), H2N2 (in 1957), and H3N2 (in 1968) subtypes have been successful in causing a pandemic so far. Further, the H1N1 and H3N2 are the predominant IAV subtypes that have been causing recurring seasonal epidemics [2]. In addition, the H17, H18, N10, and N11 subtypes have been isolated from bats [5,6]. A potential 19th HA subtype with genetic similarity to the H9 subtype has been recently detected in a duck [7,8].

## 3. H5N1 Subtype

The H5N1 subtype of IAV has become significant since 1997, when it was first discovered as a pure avian signature IAV to infect humans and cause severe respiratory disease with a high mortality rate [9,10]. Since then, H5N1 has been constantly circulating in nature with a devastating impact on the poultry industry and sporadic infections in humans [11]. Since 2020, H5N1 has had resurgent interspecies and intercontinental transmission and has been detected to infect multiple mammals, including cows, a newly discovered IAV host [12,13,14]. This review will summarize the literature generated so far on the H5N1 subtype with a focus on the latest events.

### 3.1. Emergence and Evolution of H5Nx Lineages

The H5N1 subtype was first detected in 1955 as a fowl plague virus, causing severe infection in chickens [15]. H5N1 remained active in avian populations until it was first discovered to infect humans in Hong Kong in 1997 [10,16]. Initially, H5N1 infected a child who died from a severe respiratory disease [9]. Soon after, more human cases of H5N1 were found, totaling 18, of which 6 died [10]. This H5N1 subtype was a pure avian-origin IAV and emerged from a reassortment event between a goose H5N1 subtype and other avian IAV subtypes and then jumped into humans through direct contact with infected animals [16]. No further human cases of the H5N1 subtype were discovered, but it continued to circulate in geese and other avian species and reassorted with other avian IAV subtypes, which generated various H5N1 genotypes. These H5N1 genotypes crossed over to chickens and other avian species and caused multiple disease outbreaks [17,18].

In 2003, the H5N1 subtype reappeared in humans and infected two people in southern China, of whom one died [19]. This H5N1 subtype had a different genotype but contained the same HA, albeit with antigenic variation, as the H5N1 subtype that infected humans in 1997 [19]. This H5N1 subtype then spread to other Southeast Asian countries and caused infections in poultry as well as humans during 2003–2004 [20]. In 2005, H5N1 was detected in migratory birds in China [21,22] and, subsequently, established itself in the poultry across Asia and continued to evolve genetically and antigenically [23,24]. By 2008, H5N1 spread to Africa, Europe, and the Middle East; was detected in more than 60 countries; and became endemic in poultry [25,26]. Consequently, H5N1 evolved regularly through mutations and reassortments with different NA subtypes, and the H5 HA diversified into multiple lineages and phylogenetic clades and subclades [23,27,28]. Since 2020, the H5N1 subtype clade 2.3.4.4b has become the dominant clade globally [29].

### 3.2. Emergence of Clade 2.3.4.4

The H5 clade 2.3.4.4 emerged in China in 2013 [30], but it was first detected as the H5N8 subtype in ducks in South Korea [31]. This subtype emerged through reassortment between goose H5N1 and other avian IAV subtypes [31]. The H5N8 subtype then spread intercontinentally to Europe and North America through migratory birds [32]. The H5N8 subtype was highly promiscuous, and, during the intercontinental spread, reassorted frequently with multiple NA subtypes [32,33]. By 2020, the H5 clade 2.3.4.4 became dominant and was divided into subclades ‘a-h’ [34]. Of which, the H5Nx subtypes belonging to clade 2.3.4.4b became more prevalent [35,36]. The clade 2.3.4.4b H5N1 subtype emerged through reassortment between H5N8 and other avian IAV subtypes in Europe during 2020 and then spread globally [37,38]. Since 2021, clade 2.3.4.4b H5N1 subtype has been detected worldwide in different avian and mammalian hosts and has replaced most of the other H5Nx subtypes [37].

### 3.3. Geographical Distribution of Clade 2.3.4.4b

**Figure 2:**
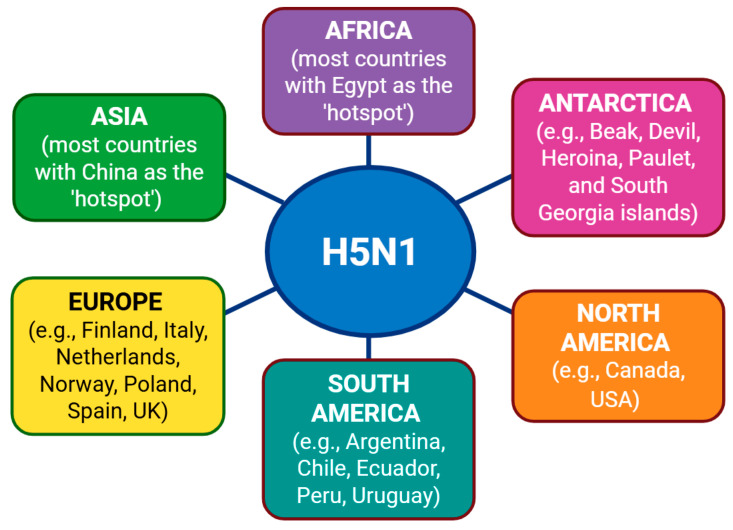
Geographical distribution of the H5N1 subtype in the world. The H5N1 subtype (indicated as blue ellipse) has been detected in almost all continents (indicated as different colored boxes), except in the Oceanic region (Australia, New Zealand, and Pacific Islands). Illustration created with BioRender.com.

Clade 2.3.4.4b H5N1 subtype was restricted to Asia until 2014 [37]. Since then, it has spread globally and has been detected in multiple hosts in Antarctica, Africa, Europe, and North and South America (Figure 2). However, this virus is yet to be detected in New Zealand and Australia, except for one imported human case of clade 2.3.2.1a in the latter [39]. In Antarctica, clade 2.3.4.4b H5N1 subtype has been detected in Beak, Devil, Heroina, Paulet, and South Georgia islands and the sub-Antarctic islands of Crozet and Kerguelen in different species of birds and marine mammals [40,41,42]. In Africa, this virus has been detected in most countries, with Egypt being the hotspot for its constant circulation and evolution in poultry [29,37,43,44,45,46,47]. In Europe, the clade 2.3.4.4b H5N1 subtype has been detected in, e.g., Finland, Italy, the Netherlands, Norway, Poland, Spain, and the UK [48,49,50,51,52,53,54,55,56,57,58]. In North America, clade 2.3.4.4b H5N1 subtype is prevalent in the USA and has been detected in humans and multiple species of domestic, farm, and wild animals [14,59,60,61,62,63,64,65,66,67,68]. In addition, the virus has also been detected in Canada [38,69,70,71]. In South America, H5N1 clade 2.3.4.4b has been detected in, e.g., Argentina, Chile, Ecuador, Peru, and Uruguay [72,73,74,75,76,77,78,79,80,81,82]. In Asia, this virus remains active and, in addition to China, has been detected in many other countries, e.g., India, Indonesia, Japan, Maldives, Mongolia, Russia, South Korea, and Sri Lanka [47,83,84,85,86,87,88,89,90,91,92,93]. The global status of H5N1 infections country-wise is regularly updated by the Center for Health Protection, Department of Health, Hong Kong Special Administrative Region [94].

### 3.4. Host Range Expansion, Including Cattle

The H5N1 subtype has been detected in various avian and mammalian species that inhabit air, land, and water (Table 1). Alarmingly, it has been discovered to infect a new mammalian host, the cow.

#### 3.4.1. Avian

Birds, both domestic and wild, are the primary host of avian IAVs. Since 2021, clade 2.3.4.4b H5N1 subtype viral RNA has been frequently detected in or infectious virus has been isolated from both domestic [68,75,86,91,95] and wild birds (Table 1). The wild birds include albatross and fulmar [41], cormorant [61,72], crane [89], crow [90], emu [85], gull and pelican [50,51,56,72,81,82,96], various raptors [60,97,98], sheathbill [40], sanderling [72,99], skua [40,41,51], swan [83,100], teal [65], tern [77], and others [45,69,84,117] (Table 1). In addition, H5N1 viral RNA has been detected in, or infectious virus has been isolated from, wetlands and water bodies [62].

#### 3.4.2. Canine

Clade 2.3.4.4b H5N1 subtype viral RNA has been detected in, or the H5N1 infectious virus has been isolated from, domestic cats in France [101], Poland [57,102], South Korea [103,104], and the USA [14,105,106,107,108] and in stray cats in the Netherlands [109] and South Korea [103]. Further, H5N1 viral RNA has been detected in wild cats in the USA [110,111], and the H5N1 infectious virus has been isolated from wild leopards in South Korea [93]. Furthermore, H5N1 viral RNA or infectious virus has been detected in foxes, raccoons, and coyotes in Canada, Finland, and the USA [52,55,56,71,111] (Table 1). In addition, H5 antibodies have been detected in domestic cats and dogs in Italy [53] and Poland [118], hunting dogs in the Netherlands [49] and the USA [119], and lynx, foxes, and wolves in Alaska, USA [63].

#### 3.4.3. Marine Mammals

Marine mammals like seals and whales serve as the spillover hosts of multiple IAV subtypes [2,120]. Therefore, marine mammals have also been exposed to the clade 2.3.4.4b H5N1 subtype (Table 1). The clade 2.3.4.4b H5N1 viral RNA or infectious virus has been detected in or isolated from different species of pinnipeds, e.g., elephant seal, fur seal, sea lion, and walrus, in Antarctica [41,42], Argentina [75,78], Canada [70], Chile [73,81], Norway [36], Peru [72], Uruguay [76,77,112], and the USA [113]. In addition, the RNA and infectious progeny of this virus have been detected in dolphins in Peru [72,114] and the USA [115] (Table 1).

#### 3.4.4. Other Mammals

Clade 2.3.4.4b H5N1 subtype has also been detected in other mammals, e.g., minks, ferrets, and bears. The RNA or antigen of this virus has been detected in minks in Canada and Finland [52,71] and Spain [48], a bear [66], skunks, opossums, and a fisher in the USA [111], and pet ferrets in Poland [116] (Table 1). Furthermore, bears in Alaska, USA [63], and horses in Mongolia [87] were seropositive for H5 and N1 antigens.

#### 3.4.5. Cows, Detection in Cow Milk, and Spillover Infection in Dairy Farm Workers

In early 2024, clade 2.3.4.4b H5N1 subtype was detected to infect dairy cows on multiple farms in the USA [14] (Table 1). In February-March 2024, dairy cows in several farms in Texas, Kansas, New Mexico, and Ohio presenting with nonspecific illness and reduced feed intake and milk production tested positive for the H5N1 subtype [121]. Later, the clade 2.3.4.4b viral RNA and infectious progeny were isolated from those cows [13,14]. Subsequently, this virus spread to other states of the USA through interstate movement of cattle and was detected in cow herds in Michigan (late March 2024), North Carolina, Colorado, and South Dakota (April 2024), Iowa, Minnesota, and Wyoming (June 2024), Oklahoma (July 2024), California (August 2024), Nebraska (September 2024), and Utah (October 2024) [122,123]. As of 17 November 2025, hundreds of dairy cow herds in 18 USA states have been reported to be affected by this virus [124].

The H5N1 infection significantly reduced the milk production in cows [125]. Importantly, the H5N1 RNA and infectious H5N1 progeny were also detected in cow milk [13,14,106,126,127] and milking equipment and surfaces [128]. Furthermore, the virus remained infectious in unpasteurized milk and milk products like cheese and whey [14,106,129]. Although pasteurization of milk inactivated the H5N1 virus, its viral RNA was detected in pasteurized milk, and this virus remained stable in milk at lower temperatures [126,127,130].

The spillover infection of clade 2.3.4.4b H5N1 subtype was also detected in dairy farm workers in different states of the USA [131,132,133]. As of 24 November 2025, 41 human H5N1 cases in the USA have been attributed to exposure to dairy herds [134]. Of these, 38 human cases were reported in California, 1 in Colorado, 2 in Michigan, 1 in Nevada, and 1 in Texas [134].

### 3.5. Transmission

As mentioned above, since its emergence in humans in 1997, the H5N1 subtype has infected multiple avian and mammalian species as well as humans on multiple continents. However, this virus has not yet acquired the capability of sustained human-to-human transmission by aerosol—a characteristic required for pandemic formation [135]. Although intra- and inter-species transmission and intercontinental spread of clade 2.3.4.4b H5N1 subtype have intensified since 2020, its major mode of transmission remains through direct contact with infected hosts.

**Figure 3:**
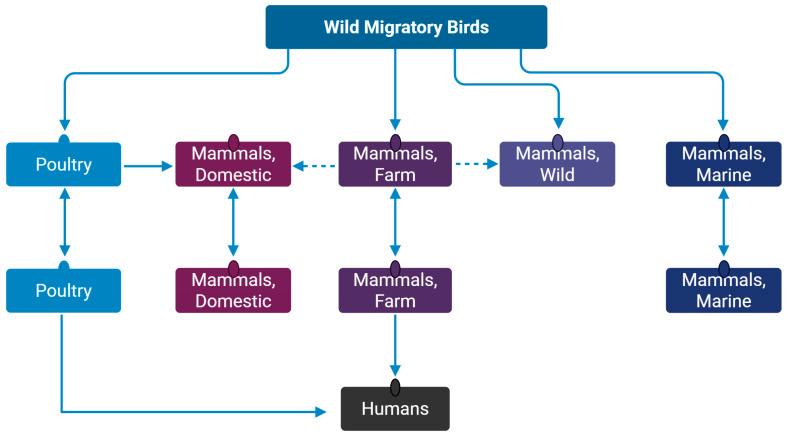
H5N1 subtype transmission in various hosts. The H5N1 subtype is prevalent in wild migratory birds, which spread this virus globally and transmitted it to poultry, farms, and wild and marine mammals. The virus then further spread among poultry, farms, and marine mammals. Subsequently, from poultry and farm mammals, this virus spilled over to domestic mammals and humans. Arrows indicate the H5N1 subtype transmission between hosts. Illustration created with BioRender.com.

The H5N1 subtype was mainly carried by wild migratory birds within and across continents [22,23,30,32,38,44,68,84,117,136,137]. From wild birds, it was transmitted to commercial and backyard poultry, which led to its spread in poultry locally [17,20,23,24,32,33,35,45,68] (Figure 3). Also, the global poultry trade may have contributed to its spread globally and then locally [138]. The marine mammals likely contracted this virus through scavenging wild birds, exposure to contaminated coastal waters, or aerosolized droplets at haul-out sites [73,77,112,113,137], and then transmitted it to other marine mammals [77,78,81,137] (Figure 3) and, occasionally, back to wild birds [77,78]. Similarly, wild mammals contracted this virus by eating the infected wild bird carcasses [55,111,139]. Wild birds also contributed to the transmission of H5N1 to farmed mammals, which led to its onward transmission in the farm [48,52,56]. Further, domestic mammals like cats received this virus by eating infected poultry or from nearby poultry farms [57,58,101,102]. The infected domestic cats may have transmitted this virus to domestic ferrets [116].

Cows also potentially contracted H5N1 from wild birds that led to cow-to-cow transmission within and between farms [13,14,122]. Also, there were indications of cow-to-cat and cow-to-raccoon transmissions [13,107]. Further, this virus spilled over to dairy farm workers through direct contact with infected cows and contaminated milking equipment or surfaces [123].

The H5N1 isolates from cows, dairy farm workers, and minks exhibited limited airborne transmission in the ferret model [80,85,133,140,141]. Furthermore, experimentally infected animals can shed this virus in the air, which can be detected in air samples [142,143]. These studies indicate that the potential of sustained aerosol transmission by the latest H5N1 isolates exists.

### 3.6. Viral Pathogenesis

#### 3.6.1. Viral Pathogenesis in Naturally Infected Hosts

The 1997 H5N1 subtype and its subsequent variants were highly pathogenic and caused severe disease, complications, and high mortality in different avian species and humans after natural infection [9,18,20,21,22,144]. The common symptoms and complications in birds were cloudy eyes, ataxia, lethargy, and paralysis [18,22], and in humans, fever (39 °C), pneumonia, acute respiratory distress syndrome (ARDS), septic shock, and renal failure [144]. As of 11 December 2025, a total of 993 human cases of infection with all variants of the H5N1 subtype have been reported since 1997, of which 477 have died [94], resulting in a mortality rate of 48%. However, the majority of these cases occurred between 1997 and 2014. Clade 2.3.4.4b H5N1 has caused sporadic infections in humans with both severe (pneumonia, septic shock) and mild (fever, respiratory symptoms—cough, sore throat, or shortness of breath) symptoms [59,67,80,131,132,145,146,147]. The dairy farm workers infected with this virus displayed mild respiratory disease symptoms and conjunctivitis [131,132]. Of 132 human H5N1 cases detected from 2020 to 11 December 2025, only 22 have died [94], which accounts for a mortality rate of just above 16%.

Clade 2.3.4.4b H5N1 caused variable disease and mortality in other host species after natural infection. Due to infection with this virus, mass mortality of different birds and marine mammals has been witnessed in the USA [60,61,99], Antarctic islands [42,148], Canada [70], Chile [73], Japan [89,90], Norway [149], Peru [72], South Korea [86], and Uruguay [76,77]. Further, several domestic, farm, and wild animals died in Canada [71], Finland [52,56], Poland [57,102,116], the USA [106,108,110], and South Korea [103]. Many of these birds and animals died with severe respiratory and/or neurological disease [52,55,57,71,72,73,77,81,98,105,106,108,111,116,150]. Further, viral RNA or antigen was detected in the lung, brain, heart, pancreas, kidney, spleen, intestine, or liver tissues of infected wild birds and animals, indicating a systemic infection and wide tissue tropism [55,73,98,111,116]. Cows infected with this virus displayed both clinical and subclinical symptoms [125]. The cows that displayed clinical symptoms exhibited decreased food intake, rumination, and milk production [13,14,125]. In infected cows, the viral RNA or antigen was mostly detected in mammary gland tissues [13,14]. Furthermore, both IAV receptors—α2,3-linked sialic acids and α2,6-linked sialic acids—are present in mammary gland tissues [151,152,153], and the H5N1 subtype replicates in ex vivo culture of bovine mammary gland tissues [154].

#### 3.6.2. Viral Pathogenesis in Experimentally Infected Hosts

The 1997 H5N1 subtype and its subsequent variants isolated from birds and humans were also highly pathogenic in experimental animal models and caused severe disease and high mortality [9,18,22]. Clade 2.3.4.4b H5N1 isolated from poultry caused a severe disease in chickens and in ducks, with ducks displaying neurologic signs [46,86,91]. However, pigeons infected with a clade 2.3.4.4b H5N1 chicken isolate did not develop clinical symptoms [155]. The clade 2.3.4.4b H5N1 subtype isolated from cows caused a systemic and lethal infection in mice and ferrets [140,156,157,158,159], severe disease in monkeys [160], and severe mastitis in goats [161]. Furthermore, clade 2.3.4.4b H5N1, isolated from a human who contracted this virus from a cow and exhibited mild symptoms, caused severe disease in mice and ferrets [162,163]. Also, a human isolate of this virus caused a lethal infection in ferrets [80]. However, in experimentally infected cows, the cow isolates of clade 2.3.4.4b H5N1 caused similar mild disease as natural infection [164,165]. Further, the viral antigen was detected in mammary gland tissues, and infected cows produced less milk and shed the virus in milk [164,165]. This indicates that milk or milking equipment and surfaces are the primary routes of viral transmission from cows.

#### 3.6.3. Molecular Determinants of Viral Pathogenesis

NS1, the main virulence factor of IAV [166], is one of the main determinants of H5N1 pathogenesis [167,168,169]. NS1 is a remarkable multifunctional protein and promotes IAV infection in multiple ways. It inhibits host gene expression and antagonizes the host’s innate immune response against IAV [166]. Since 1998, NS1 of different H5N1 strains circulating in different host species has evolved through selection that determined the pathogenicity of those H5N1 variants in different avian and mammalian hosts. These selections were a series of point mutations [170,171,172,173,174,175] and deletions [176,177,178,179,180] in different NS1 domains that affect its ability to inhibit host gene expression and antagonize host innate response.

The HA cleavage site also determines the pathogenicity of IAV. During entry to host cells, HA is cleaved by host proteases to expose the HA fusion peptide, which facilitates the fusion of the viral envelope with the host endosomal membrane and release of IAV vRNPs in the cytoplasm [181]. The presence of single or multiple basic amino acids in the HA cleavage site determines which host proteases cleave the HA and whether an IAV subtype is low pathogenic or highly pathogenic and causes a localized infection or systemic infection, respectively [182]. Cleavage sites with multiple basic amino acids, which can be recognized by ubiquitously expressed proteases like furin [182], are a characteristic of highly pathogenic avian IAV H5 and H7 subtypes [183,184]. The cleavage site in the HA of the H5N1 subtype, which emerged in 1997, possessed multiple basic amino acids [9,16,185,186], and subsequent variants of this virus maintained the multi-basic amino acid cleavage site [22,23,24]. Also, subsequent H5Nx reassortants that emerged in South Korea, Europe, and North America maintained the multi-basic amino acid cleavage site in HA [31,34,35,36,187]. Likewise, the HA of clade 2.3.4.4b H5N1 subtype maintained the multi-basic amino acid cleavage site [47,74,89,91,142,188].

### 3.7. Molecular Determinants of Host Adaptation

#### 3.7.1. Mammals

**Figure 4:**
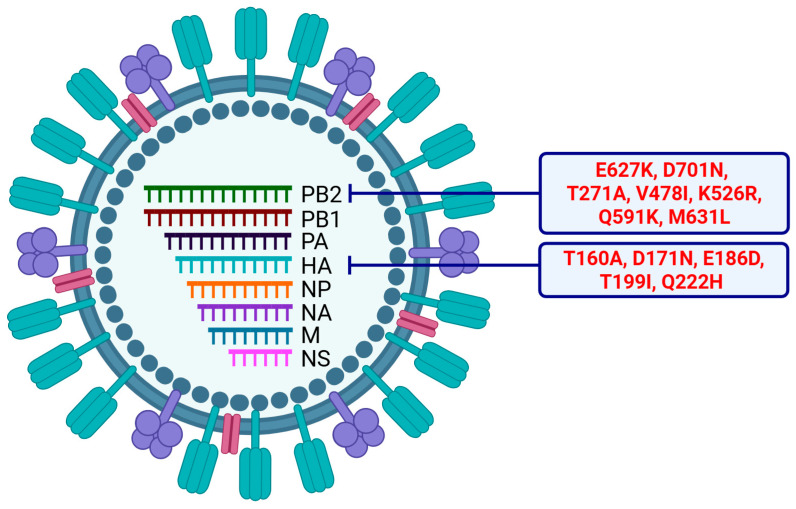
Mutations detected in the PB2 and HA genes of clade 2.3.4.4b H5N1 subtype isolates. As in Figure 1, this illustration represents an IAV H5N1 subtype virus particle. The icons in the envelope (shown as gray circle) in turquoise indicate the HA protein, in purple the NA protein, and in magenta the M2 protein. The gray dots underneath the envelope indicate M1 protein. The eight different color icons in the center indicate eight vRNPs with named gene segments. In the PB2 gene (shown in green), mammalian adaptive mutation E627K was detected in the cat [58,102,103], fox [55,71,111], human [146], mouse [190], seal [42,113], and raccoon [111,137] isolates, and mutation D701N was detected in the cat [103], fox [71], human [81], seal [77,78,81,113,137], and raccoon [111] isolates. Also, in the PB2 gene, uncommon mutations, like T271A, were detected in mink isolates [48,52,141]; V478I in ferret isolates [194]; K526R in cat isolates [58]; Q591K in seal isolates [77,78,81]; and M631L in cow isolates [140,193]. In the HA gene (shown in turquoise), mutations T160A [195], D171N [196], and T199I [197] were detected in cow isolates, and mutations E186D and Q222H in a human isolate [146]. Illustration created with BioRender.com.

Avian IAVs cannot efficiently replicate in mammals, and, to adapt to a mammalian host, they acquire adaptive mutations. These adaptive mutations primarily occur in the IAV RNA polymerase protein, PB2, and a single amino acid mutation from glutamic acid (E) to lysine (K) at position 627 of an avian IAV PB2 (Figure 4) is sufficient for its adaptation to a mammalian host [189]. Most of the mammalian and human isolates of the 1997 H5N1 subtype possessed the E627K mutation in PB2 [21,185] (Figure 4). Likewise, several mammalian isolates of clade 2.3.4.4b H5N1 subtype possess the E627K mutation [42,52,55,58,71,102,103,111,113,137,146,190] (Figure 4). In addition, some clade 2.3.4.4b H5N1 isolates from emu and duck also contain this mutation [44,85], and the emu isolate efficiently replicated and was highly pathogenic in mice [85]. Further, the clade 2.3.4.4b H5N1 isolated from red fox acquired the E627K mutation during replication in mice [191]. Interestingly, most clade 2.3.4.4b H5N1 isolates from cows did not have the E627K mutation [122,140,192], but the H5N1 isolates from dairy farm workers, who contracted this virus from cows, did acquire this mutation [132,193].

Another common mammalian adaptation mutation in PB2 is D701N [198], which many recent clade 2.3.4.4b H5N1 mammalian isolates have also acquired (Figure 4) [71,77,78,81,103,111,113,137]. In addition, some uncommon adaptive mutations have been identified in the PB2 of this virus isolated from minks (T271A) [48,52,141], ferrets (V478I) [194], cats (K526R) [58], marine mammals (Q591K) [77,78,81], and cows (M631L) [140,163,193] (Figure 4).

#### 3.7.2. Humans

To adapt to humans and acquire efficient human-to-human transmission, avian IAVs must switch the receptor specificity from α2,3-linked sialic acids to α2,6-linked sialic acids [199]. A single amino acid mutation in the HA of avian IAVs at position 226 from glutamine (Q) to leucine (L) is sufficient for their adaptation to bind α2,6-linked sialic acids [200]. The 1997 H5N1 subtype variants isolated from humans did not acquire the Q226L mutation, though they exhibited some ability to bind α2,6-linked sialic acids under experimental conditions [201,202,203]. Likewise, clade 2.3.4.4b H5N1 mammalian isolates have not acquired the Q226L mutation [156,195,204] and preferentially bind to α2,3-linked sialic acid under experimental conditions [156,195,197,205,206,207,208]. However, a 2024 report found that a cow isolate can bind to α2,6-linked sialic acids [140], but other subsequent reports found no evidence of it [209,210]. Nevertheless, a clade 2.3.4.4b H5N1 human isolate was found to contain the Q226H (Q222H in H5 numbering, Figure 4) mutation in its HA [146], though the significance of this mutation for human receptor adaptation is yet to be determined.

In addition, some clade 2.3.4.4b H5N1 isolates have acquired other mutations in their HA, such as D171N [196,204], T199I [197], E186D [146], and T160A mutations [195] (Figure 4). The D171N mutation is located at antibody binding sites and may have implications for viral evasion of host immunity [204]. The T160A mutation occurred in the HA receptor binding site of a cow isolate and resulted in the loss of glycosylation at amino acid position 158, which may enhance preference for α2,6-linked sialic acids [195]. The T199I mutation is also found in the HA of most cow isolates and is located near the receptor binding site, increasing the affinity of HA to α2,3-linked sialic acids [197].

### 3.8. Antiviral Drugs and Vaccines

#### 3.8.1. Antiviral Drugs

Currently, two classes of antiviral drugs, NA and PA inhibitors, are in use to treat the IAV infections. Both phenotypic and genotypic analyses indicated that most clade 2.3.4.4b H5N1 isolates are susceptible to both classes of drugs, and the majority of them have not acquired the mutations in their NA and PA that confer resistance to these drugs [146,147,156,162,211]. Furthermore, both human and animal patients, as well as experimental animals infected with clade 2.3.4.4b H5N1 subtype, showed improved disease outcomes after treatment with NA and/or PA inhibitors [59,107,146,147,212]. Nevertheless, some H5N1 isolates from wild birds possess the H275Y mutation in NA that confers resistance to NA inhibitors [47].

#### 3.8.2. Vaccines

The USA Food and Drug Administration (FDA) has approved three vaccines, and the European Medicines Agency (EMA) has approved six vaccines against various H5 subtypes for human use. In addition, the China State Food and Drug Administration has also approved an H5N1 vaccine [213]. However, the USA has only stockpiled these vaccines and has yet to authorize them for human use. The same is the case for Europe, except Finland has authorized vaccinating individuals with an increased risk of exposure to the H5N1 virus [214]. Similarly, Canada has also authorized vaccinating similar-risk individuals with the H5N1 vaccine [215]. Although the three H5 vaccines approved in the USA were developed against early 2000 variants of the H5N1 subtype, they were effective against the clade 2.3.4.4b H5N1 virus in trials [216,217]. Nevertheless, the WHO has been maintaining and regularly updating the list of clade 2.3.4.4b H5N1 candidate vaccine viruses (CVVs), and several new clade 2.3.4.4b H5 vaccines are in either clinical trials or development using different formulations and platforms [214]. These include an mRNA vaccine [214,218,219], live-attenuated NS1-deficient vaccine [220,221], intranasal vaccine [221,222], DNA vaccine [223,224], recombinant vaccine [225], and nanoparticle-based vaccine [226]. Of these, the ferritin-based nanoparticles represent a promising platform for the next-generation H5N1 vaccine [226]. Ferritin is a ubiquitous protein that self-assembles into nanocages, which can be utilized as carriers of viral antigens, like IAV HA. The HA-conjugated ferritin vaccines demonstrate preclinical efficacy against multiple IAV subtypes, including H5N1, and have progressed to Phase 1 clinical trials [226]. Also, several vaccines are in development or trial for vaccinating the chickens, geese, turkeys, and cattle [227,228,229,230], though some existing commercial poultry H5 vaccines protect chickens against clade 2.3.4.4b H5N1 challenge in preclinical studies [231,232]. In addition, towards developing the passive immunization regimens, several monoclonal antibodies have been identified that neutralize clade 2.3.4.4b H5N1 virus in mice [233].

### 3.9. Public Health Response and Preparedness

Since 1997, surveillance led by the WHO and local public health agencies has been the key part of the public health response to contain H5N1 spread. In addition to the WHO, the USA Centers for Disease Control and Prevention (CDC), European Centre for Disease Prevention and Control (ECDC), Centre for Health Protection (CHP), Hong Kong Special Administrative Region, and government agencies in many other countries regularly track the status of H5N1 in humans, birds, and animals. As per the CDC survey, public health authorities in all 50 USA states have the ability, albeit with variable efficiency, to monitor the epidemiology of the H5N1 virus [234]. The USA Department of Agriculture (USDA) has implemented a National Milk Testing Strategy to screen milk, and the CDC recommends screening humans working with known H5N1 animal hosts, like chickens and cows [235]. The Finnish Institute for Health and Welfare has also implemented similar guidelines for humans working on fur farms [52]. Furthermore, the CDC is offering free seasonal flu vaccination to farm workers to reduce the risk of H5N1 co-infection with seasonal IAVs and potential viral reassortments [235]. The USA Geological Survey has designed a 7-goal strategy to combat the spread of H5N1 in wildlife [236]. The South Korean Ministry of Agriculture, Food and Rural Affairs has implemented a Duck Farming Restriction Policy and, according to this, limited the duck farming during the Northern Hemisphere winter months [237].

Large-scale culling of poultry has been the main measure to control further spread of the H5N1 subtype in Europe and North America [37,238]. However, China, in addition to culling, adapted the strategy of vaccinating poultry to stop the spread of this virus [238,239,240]. Mexico is the only country in North America that is vaccinating the poultry [241]. The European Union (EU) established a legal framework in 2023 to vaccinate poultry, though only with non-live vaccine formulations [242,243]. Consequently, two H5 vaccines have been authorized to vaccinate poultry in the EU, and France has become the first EU country to mandate the vaccination of poultry [242,243]. Now, other EU countries, like Austria, are evaluating such a vaccination strategy [242].

## 4. Concluding Remarks

The influenza virus is one of the few pathogens that regularly keep the global public health agencies on their toes. The influenza virus is a primordial pathogen and will potentially exist in nature for centuries to come. Particularly, the IAV will be difficult to eradicate due to its genomic structure, ability to evolve regularly, and broad host range. Although IAV exists in multiple HA subtypes, three subtypes, H1, H3, and H5, have had a regular, significant impact on human and animal health globally. The H1 and H3 subtypes, in addition to causing the 1918, 2009, and 1967 pandemics, respectively, have been causing recurring seasonal epidemics in humans. Whereas the H5 subtype has been causing devastating outbreaks in poultry.

As is evident from this review, the IAV H5 subtypes, particularly H5N1, undoubtedly have a global presence now. Barring the Oceania region (Australia, New Zealand, and Pacific Islands), H5N1 has been detected in multiple countries on all continents. It has been infecting and killing hosts inhabiting the land, water, and air, and affecting the wildlife and marine life ecosystems. Further, this virus is exhibiting robust intraspecies transmission among different avian and marine hosts. The frequent interspecies transmission has also been occurring between hosts that inhabit near poultry farms or are exposed to migratory birds.

However, the currently circulating H5 subtypes, including the clade 2.3.4.4b H5N1 subtype, have not acquired the Q226L mutation in their HA that confers the ability of avian IAVs to bind to human receptors [244,245] and transmit among humans through aerosol. There remains a knowledge gap, and it should be the surveillance priority to determine how H5N1 subtypes will naturally acquire such mutations and the ability of aerosol transmission. Nevertheless, some clade 2.3.4.4b H5 HA have acquired other mutations near the receptor binding site, which could help the H5N1 subtype to cross the avian-to-human species barrier [146,195,197]. Furthermore, some variants of this virus have acquired the mammalian adaptive mutation E627K in their PB2. This indicates that H5N1 has the potential to acquire the avian-to-human barrier-crossing mutations.

Thus, the currently circulating H5N1 subtype has pandemic potential, though its internal gene constellation is not the same as the 1997 H5N1 subtype. The 1997 H5N1 subtype has gone through multiple reassortment events with other avian IAVs in different locations [28]. Furthermore, since 2021, clade 2.3.4.4b H5N1 variants of Eurasian, North American, and South American lineages have undergone, and continue to undergo, multiple reassortment events with other avian IAVs in wild and domestic birds, giving rise to multiple clade 2.3.4.4b H5N1 genotypes [54,68,69,122,246,247]. Consequently, the currently circulating H5N1 subtype has swapped 6 internal genes with other avian IAVs and lost some virulence and is not as lethal in humans as the 1997–2003 H5N1 variants. Another reason for the lower virulence of this H5N1 subtype in humans could be the existence of pre-existing immunity due to past IAV infections [235,248]. A recent study has found that ferrets with pre-existing immunity to 2009 pandemic H1N1 and seasonal H3N2 subtypes do not develop severe disease upon H5N1 subtype challenge [249].

Nevertheless, the H5N1 subtype remains a pandemic threat, as the clade 2.3.4.4b H5N1 subtype is now endemic in several animals globally. Further, it could adapt to ruminants, as, in addition to cows, the clade 2.3.4.4b H5N1 has been detected to infect sheep and goats [13,196,250]. Furthermore, the H5N1 subtype may reassort with swine IAV subtypes, though natural infections of pigs with this virus have been sporadic. Nevertheless, pigs are the natural host of IAV and are considered the “mixing vessel” of IAVs and have been found to be susceptible to experimental infection with different isolates of clade 2.3.4.4b H5N1 subtype [251,252,253]. Therefore, it is necessary that the H5N1 subtype remain the focus of continuous surveillance and research. The latter must include identification and pathogenic characterization of any new mutations in IAV proteins, particularly in HA and PB2. Also, it is important to assess the aerosol transmissibility of H5N1 variants with any new non-canonical mutations in their HA. Furthermore, the susceptibility of any new variants of the H5N1 subtype to available NA and PA inhibitors should be tested regularly. Accordingly, vaccine strains should be updated, and new antiviral drugs should be developed.

There should also be a global consensus on managing the H5N1 outbreaks in livestock, poultry, and wild and marine mammals and their spillovers to humans. The continuous culling of poultry is not economically sustainable [74,254] and, due to this, many poultry farm owners are reluctant to consent to H5N1 testing in their farms [235]. Furthermore, post-culling, the H5N1 virus can always transmit back to the poultry from its reservoir host, wild birds. Similarly, the indiscriminate culling of wildlife and marine mammals will lead to loss of biodiversity and disruption in ecosystems. Therefore, new, improved, and concerted One Health biosecurity measures are needed to monitor and prevent the interspecies transmission of the H5N1 subtype and the evolution and emergence of new H5Nx variants [255].

Vaccination of livestock and poultry could be a good strategy, as this strategy has been quite effective in China [238]. Some of the existing H5 vaccines used in chickens in China and elsewhere are still effective against clade 2.3.4.4b H5N1 subtype [231,232,256]. In parallel, the non-pharmaceutical control strategies (NPCS) could be practiced [257], and farming practices could be standardized and modernized. This includes reducing the farm density and proximity between farms [237,258,259,260], having onsite farm litter and vehicle decontamination facilities, and avoiding the shared dead bird disposal sites [261]. Further, the animal–human interface can be sealed by regularly vaccinating and screening the farm workers and providing them with cooling personal protective equipment (PPE) during hot weather. Furthermore, environmental screenings of wastewater, wetlands, farms, and wildlife should be carried out regularly, and any identified H5N1 subtype should be sequenced [262,263,264]. Finally, computational modeling, the use of artificial intelligence, and microbial source tracking could be employed to identify the emergence of new H5Nx variants [265,266,267].

## Figures and Tables

**Table 1:** The host range of IAV H5N1 subtype.

Host	Species
Avian	Chicken, Duck [68,75,86,91,95], Albatross [41], Cormorant [61,72], Crane [89], Crow [90], Emu [85], Fulmar [41], Gull [50,51,56,72,81,82,96], Pelican [72,81,82,96], Raptors [60,97,98], Sheathbill [40], Sanderling [72,99], Skua [40,41,51], Swan [83,100], Teal [65], and Tern [77]
Canine	Cat [14,57,101,102,103,104,105,106,107,108,109,110,111], Leopards [93], Coyote, Fox, and Raccoon [52,55,56,71,111]
Marine mammals	Pinnipeds—Elephant seal, Fur seal, Sea Lion, and Walrus [36,41,42,70,72,73,75,76,77,78,81,112,113], and Dolphin [72,114,115]
Other mammals	Cow [13,14], Human [94], Mink [48,52,71], Bear [66], Skunk, Opossum, Fisher [111], and Ferret [116]

## Data Availability

Not applicable.

## Abbreviations

The following abbreviations are used in this manuscript:

IAV	Influenza A virus
HA	Hemagglutinin
NA	Neuraminidase
WHO	World Health Organization

## References

[1] Mehta D., Spearman P., Tang Y.-W., Hindiyeh M.Y., Liu D., Sails A., Spearman P., Zhang J.-R. (2024). Influenza viruses. Molecular Medical Microbiology.

[2] Tang C.Y., Ramesh A., Wan X.-F., Tang Y.-W., Hindiyeh M.Y., Liu D., Sails A., Spearman P., Zhang J.-R. (2024). Avian and swine influenza viruses. Molecular Medical Microbiology.

[3] WHO (1979). Reconsideration of influenza A virus nomenclature: A WHO memorandum. Bull. World Health Organ..

[4] Karakus U., Sempere Borau M., Martinez-Barragan P., von Kempis J., Yildiz S., Arroyo-Fernandez L.M., Pohl M.O., Steiger J.A., Glas I., Hunziker A. (2024). MHC class II proteins mediate sialic acid independent entry of human and avian H2N2 influenza A viruses. Nat. Microbiol..

[5] Tong S., Li Y., Rivailler P., Conrardy C., Castillo D.A., Chen L.M., Recuenco S., Ellison J.A., Davis C.T., York I.A. (2012). A distinct lineage of influenza A virus from bats. Proc. Natl. Acad. Sci. USA.

[6] Tong S., Zhu X., Li Y., Shi M., Zhang J., Bourgeois M., Yang H., Chen X., Recuenco S., Gomez J. (2013). New world bats harbor diverse influenza A viruses. PLoS Pathog..

[7] Fereidouni S., Starick E., Karamendin K., Genova C.D., Scott S.D., Khan Y., Harder T., Kydyrmanov A. (2023). Genetic characterization of a new candidate hemagglutinin subtype of influenza A viruses. Emerg. Microbes Infect..

[8] Karakus U., Mena I., Kottur J., El Zahed S.S., Seoane R., Yildiz S., Chen L., Plancarte M., Lindsay L., Halpin R. (2024). H19 influenza A virus exhibits species-specific MHC class II receptor usage. Cell Host Microbe.

[9] Subbarao K., Klimov A., Katz J., Regnery H., Lim W., Hall H., Perdue M., Swayne D., Bender C., Huang J. (1998). Characterization of an avian influenza A (H5N1) virus isolated from a child with a fatal respiratory illness. Science.

[10] Centers for Disease Control and Prevention (1997). Isolation of avian influenza A(H5N1) viruses from humans—Hong Kong, May-December 1997. MMWR Morb. Mortal. Wkly. Rep..

[11] WHO Cumulative Number of Confirmed Human Cases for Avian Influenza A(H5N1) Reported to WHO, 2003–2025. https://www.who.int/teams/global-influenza-programme/avian-influenza/avian-a-h5n1-virus.

[12] WAHIS World Organization for Animal Health, Animal Disease Events. https://wahis.woah.org/#/event-management.

[13] Caserta L.C., Frye E.A., Butt S.L., Laverack M., Nooruzzaman M., Covaleda L.M., Thompson A.C., Koscielny M.P., Cronk B., Johnson A. (2024). Spillover of highly pathogenic avian influenza H5N1 virus to dairy cattle. Nature.

[14] Burrough E.R., Magstadt D.R., Petersen B., Timmermans S.J., Gauger P.C., Zhang J., Siepker C., Mainenti M., Li G., Thompson A.C. (2024). Highly Pathogenic Avian Influenza A(H5N1) Clade 2.3.4.4b Virus Infection in Domestic Dairy Cattle and Cats, United States, 2024. Emerg. Infect. Dis..

[15] Criado M.F., Caceres C.J., Castellanos I., Kidd M., Dridi S. (2025). One Hundred Forty-Seven Years Later: The Avian Influenza Virus H5N1 Goes Wild. J. Nutr..

[16] Xu X., Subbarao K., Cox N.J., Guo Y. (1999). Genetic characterization of the pathogenic influenza A/Goose/Guangdong/1/96 (H5N1) virus: Similarity of its hemagglutinin gene to those of H5N1 viruses from the 1997 outbreaks in Hong Kong. Virology.

[17] Guan Y., Peiris J.S., Lipatov A.S., Ellis T.M., Dyrting K.C., Krauss S., Zhang L.J., Webster R.G., Shortridge K.F. (2002). Emergence of multiple genotypes of H5N1 avian influenza viruses in Hong Kong SAR. Proc. Natl. Acad. Sci. USA.

[18] Sturm-Ramirez K.M., Ellis T., Bousfield B., Bissett L., Dyrting K., Rehg J.E., Poon L., Guan Y., Peiris M., Webster R.G. (2004). Reemerging H5N1 influenza viruses in Hong Kong in 2002 are highly pathogenic to ducks. J. Virol..

[19] Guan Y., Poon L.L., Cheung C.Y., Ellis T.M., Lim W., Lipatov A.S., Chan K.H., Sturm-Ramirez K.M., Cheung C.L., Leung Y.H. (2004). H5N1 influenza: A protean pandemic threat. Proc. Natl. Acad. Sci. USA.

[20] Li K.S., Guan Y., Wang J., Smith G.J., Xu K.M., Duan L., Rahardjo A.P., Puthavathana P., Buranathai C., Nguyen T.D. (2004). Genesis of a highly pathogenic and potentially pandemic H5N1 influenza virus in eastern Asia. Nature.

[21] Chen H., Smith G.J., Zhang S.Y., Qin K., Wang J., Li K.S., Webster R.G., Peiris J.S., Guan Y. (2005). Avian flu: H5N1 virus outbreak in migratory waterfowl. Nature.

[22] Liu J., Xiao H., Lei F., Zhu Q., Qin K., Zhang X.W., Zhang X.L., Zhao D., Wang G., Feng Y. (2005). Highly pathogenic H5N1 influenza virus infection in migratory birds. Science.

[23] Chen H., Smith G.J., Li K.S., Wang J., Fan X.H., Rayner J.M., Vijaykrishna D., Zhang J.X., Zhang L.J., Guo C.T. (2006). Establishment of multiple sublineages of H5N1 influenza virus in Asia: Implications for pandemic control. Proc. Natl. Acad. Sci. USA.

[24] Smith G.J., Fan X.H., Wang J., Li K.S., Qin K., Zhang J.X., Vijaykrishna D., Cheung C.L., Huang K., Rayner J.M. (2006). Emergence and predominance of an H5N1 influenza variant in China. Proc. Natl. Acad. Sci. USA.

[25] WHO/OIE/FAO H5N1 Evolution Working Group (2008). Toward a unified nomenclature system for highly pathogenic avian influenza virus (H5N1). Emerg. Infect. Dis..

[26] WHO/OIE/FAO H5N1 Evolution Working Group (2012). Continued evolution of highly pathogenic avian influenza A (H5N1): Updated nomenclature. Influenza Other Respir. Viruses.

[27] Smith G.J., Donis R.O., World Health Organization/World Organisation for Animal Health/Food and Agriculture Organization (WHO/OIE/FAO) H5 Evolution Working Group (2015). Nomenclature updates resulting from the evolution of avian influenza A(H5) virus clades 2.1.3.2a, 2.2.1, and 2.3.4 during 2013–2014. Influenza Other Respir. Viruses.

[28] Watanabe Y., Ibrahim M.S., Suzuki Y., Ikuta K. (2012). The changing nature of avian influenza A virus (H5N1). Trends Microbiol..

[29] Peacock T.P., Moncla L., Dudas G., VanInsberghe D., Sukhova K., Lloyd-Smith J.O., Worobey M., Lowen A.C., Nelson M.I. (2025). The global H5N1 influenza panzootic in mammals. Nature.

[30] Lee D.H., Torchetti M.K., Winker K., Ip H.S., Song C.S., Swayne D.E. (2015). Intercontinental Spread of Asian-Origin H5N8 to North America through Beringia by Migratory Birds. J. Virol..

[31] Lee Y.J., Kang H.M., Lee E.K., Song B.M., Jeong J., Kwon Y.K., Kim H.R., Lee K.J., Hong M.S., Jang I. (2014). Novel reassortant influenza A(H5N8) viruses, South Korea, 2014. Emerg. Infect. Dis..

[32] Global Consortium for H5N8 and Related Influenza Viruses (2016). Role for migratory wild birds in the global spread of avian influenza H5N8. Science.

[33] Ip H.S., Dusek R.J., Bodenstein B., Torchetti M.K., DeBruyn P., Mansfield K.G., DeLiberto T., Sleeman J.M. (2016). High Rates of Detection of Clade 2.3.4.4 Highly Pathogenic Avian Influenza H5 Viruses in Wild Birds in the Pacific Northwest During the Winter of 2014–15. Avian Dis..

[34] Engelsma M., Heutink R., Harders F., Germeraad E.A., Beerens N. (2022). Multiple Introductions of Reassorted Highly Pathogenic Avian Influenza H5Nx Viruses Clade 2.3.4.4b Causing Outbreaks in Wild Birds and Poultry in The Netherlands, 2020–2021. Microbiol. Spectr..

[35] Lewis N.S., Banyard A.C., Whittard E., Karibayev T., Al Kafagi T., Chvala I., Byrne A., Meruyert Akberovna S., King J., Harder T. (2021). Emergence and spread of novel H5N8, H5N5 and H5N1 clade 2.3.4.4 highly pathogenic avian influenza in 2020. Emerg. Microbes Infect..

[36] Postel A., Gremmel N., Lydersen C., Kovacs K.M., Schick L.A., Siebert U., Nymo I.H., Becher P. (2025). Highly pathogenic avian influenza virus (H5N5) detected in an Atlantic walrus (*Odobenus rosmarus rosmarus*) in the Svalbard Archipelago, Norway, 2023. Emerg. Microbes Infect..

[37] Xie R., Edwards K.M., Wille M., Wei X., Wong S.S., Zanin M., El-Shesheny R., Ducatez M., Poon L.L.M., Kayali G. (2023). The episodic resurgence of highly pathogenic avian influenza H5 virus. Nature.

[38] Caliendo V., Lewis N.S., Pohlmann A., Baillie S.R., Banyard A.C., Beer M., Brown I.H., Fouchier R.A.M., Hansen R.D.E., Lameris T.K. (2022). Transatlantic spread of highly pathogenic avian influenza H5N1 by wild birds from Europe to North America in 2021. Sci. Rep..

[39] Kumar P., Sharma A., Apostolopoulos V., Gaidhane A.M., Satapathy P. (2024). Australia’s first human case of H5N1 and the current H7 poultry outbreaks: Implications for public health and biosecurity measures. Lancet Reg. Health West. Pac..

[40] Aguado B., Begeman L., Gunther A., Iervolino M., Soto F., Vanstreels R.E.T., Reade A., Coerper A., Wallis B., Alcami A. (2024). Searching for high pathogenicity avian influenza virus in Antarctica. Nat. Microbiol..

[41] Banyard A.C., Bennison A., Byrne A.M.P., Reid S.M., Lynton-Jenkins J.G., Mollett B., De Silva D., Peers-Dent J., Finlayson K., Hall R. (2024). Detection and spread of high pathogenicity avian influenza virus H5N1 in the Antarctic Region. Nat. Commun..

[42] Clessin A., Briand F.X., Tornos J., Lejeune M., De Pasquale C., Fischer R., Souchaud F., Hirchaud E., Hong S.L., Bralet T. (2025). Circumpolar spread of avian influenza H5N1 to southern Indian Ocean islands. Nat. Commun..

[43] Fusaro A., Zecchin B., Vrancken B., Abolnik C., Ademun R., Alassane A., Arafa A., Awuni J.A., Couacy-Hymann E., Coulibaly M.B. (2019). Disentangling the role of Africa in the global spread of H5 highly pathogenic avian influenza. Nat. Commun..

[44] Olawuyi K., Orole O., Meseko C., Monne I., Shittu I., Bianca Z., Fusaro A., Inuwa B., Akintola R., Ibrahim J. (2024). Detection of clade 2.3.4.4 highly pathogenic avian influenza H5 viruses in healthy wild birds in the Hadeji-Nguru wetland, Nigeria 2022. Influenza Other Respir. Viruses.

[45] Atuman Y.J., Inuwa B., Bakam J., Orioko E., Ikemefuna N., Emmanuel H.S., Meseko C. (2025). Exotic and aquatic zoological birds harbour highly pathogenic Avian Influenza Virus, 2021: Continuous threat to poultry production, food security and public health in Nigeria. Emerg. Anim. Species.

[46] Kamel M.N., Moatasim Y., Aboulhoda B.E., Gomaa M., El Taweel A., Kutkat O., El Sayes M., GabAllah M., AbdAllah H., Gabre R.M. (2025). Genetic Characterization and Pathogenesis of Highly Pathogenic Avian Influenza Virus A (H5N1) Isolated in Egypt During 2021–2023. Viruses.

[47] Wannigama D.L., Amarasiri M., Phattharapornjaroen P., Hurst C., Modchang C., Besa J.J.V., Miyanaga K., Cui L., Fernandez S., Huang A.T. (2025). Surveillance of avian influenza through bird guano in remote regions of the global south to uncover transmission dynamics. Nat. Commun..

[48] Aguero M., Monne I., Sanchez A., Zecchin B., Fusaro A., Ruano M.J., Del Valle Arrojo M., Fernandez-Antonio R., Souto A.M., Tordable P. (2023). Highly pathogenic avian influenza A(H5N1) virus infection in farmed minks, Spain, October 2022. Euro Surveill..

[49] Duijvestijn M., Schuurman N., Vernooij J.C.M., Broekhuizen M.J., de Bruin E., Carriere B.S., van den Brand J.M.A., Wagenaar J.A., van Kuppeveld F.J.M., Egberink H.F. (2025). Hunting-training dogs and companion dogs in the Netherlands are frequently exposed to highly pathogenic avian influenza (HPAI H5) and human H1N1 virus, 2021–2023. One Health.

[50] Indykiewicz P., Przymencki M., Minias P., Jakubas D., Litwiniak K., Zielinski P., Janiszewski T., Wlodarczyk R., Ledwon M., Nowakowski J. (2025). Impact of highly pathogenic avian influenza virus (HPAIV) on Black-headed Gulls Chroicocephalus ridibundus population in Poland in 2023. Avian Pathol..

[51] Lean F.Z.X., Falchieri M., Furman N., Tyler G., Robinson C., Holmes P., Reid S.M., Banyard A.C., Brown I.H., Man C. (2024). Highly pathogenic avian influenza virus H5N1 infection in skua and gulls in the United Kingdom, 2022. Vet. Pathol..

[52] Lindh E., Lounela H., Ikonen N., Kantala T., Savolainen-Kopra C., Kauppinen A., Osterlund P., Kareinen L., Katz A., Nokireki T. (2023). Highly pathogenic avian influenza A(H5N1) virus infection on multiple fur farms in the South and Central Ostrobothnia regions of Finland, July 2023. Euro Surveill..

[53] Moreno A., Bonfante F., Bortolami A., Cassaniti I., Caruana A., Cottini V., Cereda D., Farioli M., Fusaro A., Lavazza A. (2023). Asymptomatic infection with clade 2.3.4.4b highly pathogenic avian influenza A(H5N1) in carnivore pets, Italy, April 2023. Euro Surveill..

[54] Van Borm S., Ahrens A.K., Bachofen C., Banyard A.C., Boe C.A., Briand F.X., Dirbakova Z., Engelsma M., Fusaro A., Germeraad E. (2025). Genesis and Spread of Novel Highly Pathogenic Avian Influenza A(H5N1) Clade 2.3.4.4b Virus Genotype EA-2023-DG Reassortant, Western Europe. Emerg. Infect. Dis..

[55] Bordes L., Vreman S., Heutink R., Roose M., Venema S., Pritz-Verschuren S.B.E., Rijks J.M., Gonzales J.L., Germeraad E.A., Engelsma M. (2023). Highly Pathogenic Avian Influenza H5N1 Virus Infections in Wild Red Foxes (*Vulpes vulpes*) Show Neurotropism and Adaptive Virus Mutations. Microbiol. Spectr..

[56] Kareinen L., Tammiranta N., Kauppinen A., Zecchin B., Pastori A., Monne I., Terregino C., Giussani E., Kaarto R., Karkamo V. (2024). Highly pathogenic avian influenza A(H5N1) virus infections on fur farms connected to mass mortalities of black-headed gulls, Finland, July to October 2023. Euro Surveill..

[57] Szalus-Jordanow O., Golke A., Dzieciatkowski T., Chrobak-Chmiel D., Rzewuska M., Czopowicz M., Sapierzynski R., Kardas M., Biernacka K., Mickiewicz M. (2023). A Fatal A/H5N1 Avian Influenza Virus Infection in a Cat in Poland. Microorganisms.

[58] Domanska-Blicharz K., Swieton E., Swiatalska A., Monne I., Fusaro A., Tarasiuk K., Wyrostek K., Stys-Fijol N., Giza A., Pietruk M. (2023). Outbreak of highly pathogenic avian influenza A(H5N1) clade 2.3.4.4b virus in cats, Poland, June to July 2023. Euro Surveill..

[59] Garg S., Reinhart K., Couture A., Kniss K., Davis C.T., Kirby M.K., Murray E.L., Zhu S., Kraushaar V., Wadford D.A. (2025). Highly Pathogenic Avian Influenza A(H5N1) Virus Infections in Humans. N. Engl. J. Med..

[60] Cunningham M.W., Brown J., Hardman R., Loerzel S., Kluever B.M., Zachariah T.T., Donnelly K.A., Poulson R.L., Nemeth N.M., Van Why K. (2025). Outbreaks of Highly Pathogenic H5N1 Influenza A Virus infection in Black Vultures (*Coragyps atratus*), USA, 2022. J. Wildl. Dis..

[61] Harvey J., Sullivan J.D., Poulson R.L., Carter D.L., Driscoll C.P., McGowan P.C., Callahan C.R., O’Donnell A.W., Mullinax J.M., Stallknecht D.E. (2025). Highly Pathogenic Avian Influenza Virus H5N1 in Double-crested Cormorants (*Nannopterum auritum*) of the Chesapeake Bay, USA. J. Wildl. Dis..

[62] Hubbard L.E., Givens C.E., Stelzer E.A., Killian M.L., Kolpin D.W., Szablewski C.M., Poulson R.L. (2023). Environmental Surveillance and Detection of Infectious Highly Pathogenic Avian Influenza Virus in Iowa Wetlands. Environ. Sci. Technol. Lett..

[63] Ramey A.M., Beckmen K.B., Saalfeld D.T., Nicholson K.L., Mangipane B.A., Scott L.C., Stallknecht D.E., Poulson R.L. (2025). Exposure of Wild Mammals to Influenza A(H5N1) Virus, Alaska, USA, 2020–2023. Emerg. Infect. Dis..

[64] Rubin E.J., Baden L.R., Weaver L.M., Morrissey S. (2025). NEJM Outbreaks Update—H5N1 in Indiana. N. Engl. J. Med..

[65] Stallknecht D.E., Carter D.L., Sullivan-Brugger L., Link P., Ferraro E., McCarty C., Davis B., Knutsen L., Graham J., Poulson R.L. (2024). Highly Pathogenic H5N1 Influenza A Virus (IAV) in Blue-Winged Teal in the Mississippi Flyway Is Following the Historic Seasonal Pattern of Low-Pathogenicity IAV in Ducks. Pathogens.

[66] Stimmelmayr R., Rotstein D., Torchetti M.K., Gerlach R. (2024). Highly Pathogenic Avian Influenza Virus A(H5N1) Clade 2.3.4.4b Infection in Free-Ranging Polar Bear, Alaska, USA. Emerg. Infect. Dis..

[67] Tobolowsky F.A., Morris E., Castro L., Schaff T., Jacinto M., Clement J.P., Levine M.Z., Frederick J.C., Liu F., Holiday C. (2025). Highly Pathogenic Avian Influenza A(H5N1) Virus Infection in a Child with No Known Exposure—San Francisco, California, December 2024–January 2025. MMWR Morb. Mortal. Wkly. Rep..

[68] Youk S., Torchetti M.K., Lantz K., Lenoch J.B., Killian M.L., Leyson C., Bevins S.N., Dilione K., Ip H.S., Stallknecht D.E. (2023). H5N1 highly pathogenic avian influenza clade 2.3.4.4b in wild and domestic birds: Introductions into the United States and reassortments, December 2021–April 2022. Virology.

[69] Himsworth C.G., Caleta J.M., Jassem A.N., Yang K.C., Zlosnik J.E.A., Tyson J.R., Wilson L., Kuchinski K.S., Giacinti J., Willie M. (2025). Highly Pathogenic Avian Influenza A(H5N1) in Wild Birds and a Human, British Columbia, Canada, 2024. Emerg. Infect. Dis..

[70] Lair S., Quesnel L., Signore A.V., Delnatte P., Embury-Hyatt C., Nadeau M.S., Lung O., Ferrell S.T., Michaud R., Berhane Y. (2024). Outbreak of Highly Pathogenic Avian Influenza A(H5N1) Virus in Seals, St. Lawrence Estuary, Quebec, Canada. Emerg. Infect. Dis..

[71] Alkie T.N., Cox S., Embury-Hyatt C., Stevens B., Pople N., Pybus M.J., Xu W., Hisanaga T., Suderman M., Koziuk J. (2023). Characterization of neurotropic HPAI H5N1 viruses with novel genome constellations and mammalian adaptive mutations in free-living mesocarnivores in Canada. Emerg. Microbes Infect..

[72] Leguia M., Garcia-Glaessner A., Munoz-Saavedra B., Juarez D., Barrera P., Calvo-Mac C., Jara J., Silva W., Ploog K., Amaro L. (2023). Highly pathogenic avian influenza A (H5N1) in marine mammals and seabirds in Peru. Nat. Commun..

[73] Ulloa M., Fernandez A., Ariyama N., Colom-Rivero A., Rivera C., Nunez P., Sanhueza P., Johow M., Araya H., Torres J.C. (2023). Mass mortality event in South American sea lions (*Otaria flavescens*) correlated to highly pathogenic avian influenza (HPAI) H5N1 outbreak in Chile. Vet. Q..

[74] Jimenez-Bluhm P., Siegers J.Y., Tan S., Sharp B., Freiden P., Johow M., Orozco K., Ruiz S., Baumberger C., Galdames P. (2023). Detection and phylogenetic analysis of highly pathogenic A/H5N1 avian influenza clade 2.3.4.4b virus in Chile, 2022. Emerg. Microbes Infect..

[75] Artuso M.C., Marchione V.D., Benedetti E., Bonastre P., Alvarez A.M., Piccini L., Ponde A., Barrios Benito E., Fabeiro M., Waisman K. (2025). Detection and characterization of highly pathogenic avian influenza A (H5N1) clade 2.3.4.4b virus circulating in Argentina in 2023. Rev. Argent. Microbiol..

[76] Szteren D., Franco-Trecu V. (2024). Incidence of highly pathogenic avian influenza H5N1 in pinnipeds in Uruguay. Dis. Aquat. Organ..

[77] Tomas G., Marandino A., Panzera Y., Rodriguez S., Wallau G.L., Dezordi F.Z., Perez R., Bassetti L., Negro R., Williman J. (2024). Highly pathogenic avian influenza H5N1 virus infections in pinnipeds and seabirds in Uruguay: Implications for bird-mammal transmission in South America. Virus Evol..

[78] Uhart M.M., Vanstreels R.E.T., Nelson M.I., Olivera V., Campagna J., Zavattieri V., Lemey P., Campagna C., Falabella V., Rimondi A. (2024). Epidemiological data of an influenza A/H5N1 outbreak in elephant seals in Argentina indicates mammal-to-mammal transmission. Nat. Commun..

[79] Bruno A., Alfaro-Nunez A., de Mora D., Armas R., Olmedo M., Garces J., Vaca M.S., De la Torre E., Jarrin D., Burbano L. (2023). Phylogenetic analysis reveals that the H5N1 avian influenza A outbreak in poultry in Ecuador in November 2022 is associated with the highly pathogenic clade 2.3.4.4b. Int. J. Infect. Dis..

[80] Pulit-Penaloza J.A., Brock N., Belser J.A., Sun X., Pappas C., Kieran T.J., Basu Thakur P., Zeng H., Cui D., Frederick J. (2024). Highly pathogenic avian influenza A(H5N1) virus of clade 2.3.4.4b isolated from a human case in Chile causes fatal disease and transmits between co-housed ferrets. Emerg. Microbes Infect..

[81] Pardo-Roa C., Nelson M.I., Ariyama N., Aguayo C., Almonacid L.I., Gonzalez-Reiche A.S., Munoz G., Ulloa M., Avila C., Navarro C. (2025). Cross-species and mammal-to-mammal transmission of clade 2.3.4.4b highly pathogenic avian influenza A/H5N1 with PB2 adaptations. Nat. Commun..

[82] Ariyama N., Pardo-Roa C., Munoz G., Aguayo C., Avila C., Mathieu C., Almonacid L.I., Medina R.A., Brito B., Johow M. (2023). Highly Pathogenic Avian Influenza A(H5N1) Clade 2.3.4.4b Virus in Wild Birds, Chile. Emerg. Infect. Dis..

[83] Xu Y., Wu S., Ji L., Hu J., Wang J., Li K., Yuan Y., Li G., Zhu G., Hua P. (2025). Black swans as sentinel species for the emergence of clade 2.3.4.4b highly pathogenic avian influenza (H5N1) in Shanghai, China, 2024. Virol. Sin..

[84] Kim J.Y., Jeong S., Kim D.W., Lee D.W., Lee D.H., Kim D., Kwon J.H. (2024). Genomic epidemiology of highly pathogenic avian influenza A (H5N1) virus in wild birds in South Korea during 2021–2022: Changes in viral epidemic patterns. Virus Evol..

[85] Marchenko V.Y., Panova A.S., Kolosova N.P., Gudymo A.S., Svyatchenko S.V., Danilenko A.V., Vasiltsova N.N., Egorova M.L., Onkhonova G.S., Zhestkov P.D. (2024). Characterization of H5N1 avian influenza virus isolated from bird in Russia with the E627K mutation in the PB2 protein. Sci. Rep..

[86] Cha R.M., Park M.J., Baek Y.G., Lee Y.N., Jang Y., Kang Y.M., Heo G.B., An S.H., Lee K.N., Kim J.K. (2025). Genetic characteristics and pathogenesis of clade 2.3.4.4b H5N1 high pathogenicity avian influenza virus isolated from poultry in South Korea, 2022–2023. Virus Res..

[87] Damdinjav B., Raveendran S., Mojsiejczuk L., Ankhanbaatar U., Yang J., Sadeyen J.R., Iqbal M., Perez D.R., Rajao D.S., Park A. (2025). Evidence of Influenza A(H5N1) Spillover Infections in Horses, Mongolia. Emerg. Infect. Dis..

[88] Mine J., Takadate Y., Kumagai A., Sakuma S., Tsunekuni R., Miyazawa K., Uchida Y. (2024). Genetics of H5N1 and H5N8 High-Pathogenicity Avian Influenza Viruses Isolated in Japan in Winter 2021–2022. Viruses.

[89] Esaki M., Okuya K., Tokorozaki K., Haraguchi Y., Hasegawa T., Ozawa M. (2025). Highly Pathogenic Avian Influenza A(H5N1) Outbreak in Endangered Cranes, Izumi Plain, Japan, 2022–2023. Emerg. Infect. Dis..

[90] Isoda N., Hiono T., Hew Y.L., Takaya F., Nguyen B.L., Kobayashi D., Fujino K., Sakoda Y. (2025). Dynamics of high pathogenicity avian influenza virus infection with multiple introductions in a crow flock in an urban park in Hokkaido, Japan. Comp. Immunol. Microbiol. Infect. Dis..

[91] Nishiura H., Kumagai A., Maeda M.H., Takadate Y., Sakuma S., Tsunekuni R., Mine J., Uchida Y., Miyazawa K. (2025). Pathogenic and Antigenic Analyses of H5N1 High Pathogenicity Avian Influenza Virus Isolated in the 2022/2023 Season From Poultry Farms in Izumi City, Japan. Transbound. Emerg. Dis..

[92] Pawar S.D., Tare D.S., Keng S.S., Walimbe A.M., Sharma V., Misra G., Kurkure N., Kumar N. (2025). Tracing the possible origins of the clade 2.3.4.4b highly pathogenic avian influenza H5Nx viruses from India. Virology.

[93] Si Y.J., Lee S.H., Kim D.J., Lee K., Lee M.A., Lee D.Y., Seo Y.R., Jeong H., Lee S., Lee D.H. (2025). First detection of clade 2.3.4.4b H5N1 highly pathogenic avian influenza virus in a wild leopard cat (*Prionailurus bengalensis*) in South Korea. Front. Vet. Sci..

[94] CHP H.K. Global Statistics of Avian Influenza A(H5N1). http://www.info.gov.hk/info/flu/eng/global.htm.

[95] Ssematimba A., Malladi S., Bonney P.J., St Charles K.M., Hutchinson H.C., Schoenbaum M., Marusak R., Culhane M.R., Cardona C.J. (2024). Estimating the time of Highly Pathogenic Avian Influenza virus introduction into United States poultry flocks during the 2022/24 epizootic. PLoS ONE.

[96] Castro-Sanguinetti G.R., Gonzalez-Veliz R., Callupe-Leyva A., Apaza-Chiara A.P., Jara J., Silva W., Icochea E., More-Bayona J.A. (2024). Highly pathogenic avian influenza virus H5N1 clade 2.3.4.4b from Peru forms a monophyletic group with Chilean isolates in South America. Sci. Rep..

[97] Gunther A., Krone O., Globig A., Pohlmann A., King J., Fast C., Grund C., Hennig C., Herrmann C., Piro S. (2024). Avian raptors are indicator species and victims of high pathogenicity avian influenza virus HPAIV H5N1 (clade 2.3.4.4b) in Germany. Sci. Rep..

[98] Wunschmann A., Franzen-Klein D., Torchetti M., Confeld M., Carstensen M., Hall V. (2024). Lesions and viral antigen distribution in bald eagles, red-tailed hawks, and great horned owls naturally infected with H5N1 clade 2.3.4.4b highly pathogenic avian influenza virus. Vet. Pathol..

[99] Andreasen V.A., Phillips E.G., O’Reilly A.M., Stilz C.R., Poulson R.L., Boettcher R., Tracey J.K., Nemeth N.M. (2025). Clade 2.3.4.4b Highly Pathogenic Avian Influenza H5N1 Pathology in a Common Shorebird Species (Sanderling; *Calidris alba*) in Virginia, USA. Animals.

[100] Yang J., Zhang C., Yuan Y., Sun J., Lu L., Sun H., Sun H., Chu D., Qin S., Chen J. (2023). Novel Avian Influenza Virus (H5N1) Clade 2.3.4.4b Reassortants in Migratory Birds, China. Emerg. Infect. Dis..

[101] Briand F.X., Souchaud F., Pierre I., Beven V., Hirchaud E., Herault F., Planel R., Rigaudeau A., Bernard-Stoecklin S., Van der Werf S. (2023). Highly Pathogenic Avian Influenza A(H5N1) Clade 2.3.4.4b Virus in Domestic Cat, France, 2022. Emerg. Infect. Dis..

[102] Rabalski L., Milewska A., Pohlmann A., Gackowska K., Lepionka T., Szczepaniak K., Swiatalska A., Sieminska I., Arent Z., Beer M. (2023). Emergence and potential transmission route of avian influenza A (H5N1) virus in domestic cats in Poland, June 2023. Euro Surveill..

[103] Kang Y.M., Heo G.B., An S.H., Lee H., Park E., Cha R.M., Jang Y.Y., Sagong M., Kim A.Y., Kim J. (2024). Highly Pathogenic Avian Influenza A(H5N1) Virus Infection in Cats, South Korea, 2023. Emerg. Infect. Dis..

[104] Lee K., Yeom M., Vu T.T.H., Do H.Q., Na W., Lee M., Jeong D.G., Cheon D.S., Song D. (2024). Characterization of highly pathogenic avian influenza A (H5N1) viruses isolated from cats in South Korea, 2023. Emerg. Microbes Infect..

[105] Chen C., Naru A., Mareddy V.R., Lanka S., Olmstead C., Revindran-Stam V., Sherman M., Yee H., Loose N., Delaney M.A. (2025). Highly pathogenic avian influenza A H5N1 virus infection in an immunocompromised domestic cat. ASM Case Rep..

[106] Frye E.A., Nooruzzaman M., Cronk B., Laverack M., de Oliveira P.S.B., Caserta L.C., Lejeune M., Diel D.G. (2025). Isolation of Highly Pathogenic Avian Influenza A(H5N1) Virus from Cat Urine after Raw Milk Ingestion, United States. Emerg. Infect. Dis..

[107] Gomez J.F., Bemis I.G., Shittu I., Gray G.C., Coleman K.K. (2025). Outbreak of highly pathogenic avian influenza a(H5N1) among house cats: A case series involving oseltamivir treatment. One Health.

[108] Sillman S.J., Drozd M., Loy D., Harris S.P. (2023). Naturally occurring highly pathogenic avian influenza virus H5N1 clade 2.3.4.4b infection in three domestic cats in North America during 2023. J. Comp. Pathol..

[109] Duijvestijn M., Schuurman N., Vernooij J.C.M., van Leeuwen M., van den Brand J.M.A., Wagenaar J.A., van Kuppeveld F.J.M., Egberink H.F., de Haan C.A.M., Verhagen J.H. (2024). Highly pathogenic avian influenza (HPAI) H5 virus exposure in domestic cats and rural stray cats, the Netherlands, October 2020 to June 2023. Euro Surveill..

[110] Turner H.M., Fuller A.K., Twining J.P., Hitchener G.R., Fadden M.A., Stallknecht D.E., Poulson R.L., Carter D.L., Watson M.B., Schuler K.L. (2025). Highly Pathogenic Avian Influenza Virus Exposure and Infection in Free-Ranging Bobcats (*Lynx rufus*) in New York, USA. J. Wildl. Dis..

[111] Elsmo E.J., Wunschmann A., Beckmen K.B., Broughton-Neiswanger L.E., Buckles E.L., Ellis J., Fitzgerald S.D., Gerlach R., Hawkins S., Ip H.S. (2023). Highly Pathogenic Avian Influenza A(H5N1) Virus Clade 2.3.4.4b Infections in Wild Terrestrial Mammals, United States, 2022. Emerg. Infect. Dis..

[112] Paz M., Franco-Trecu V., Szteren D., Costabile A., Portela C., Bruno A., Moratorio G., Moreno P., Cristina J. (2024). Understanding the emergence of highly pathogenic avian influenza A virus H5N1 in pinnipeds: An evolutionary approach. Virus Res..

[113] Puryear W., Sawatzki K., Hill N., Foss A., Stone J.J., Doughty L., Walk D., Gilbert K., Murray M., Cox E. (2023). Highly Pathogenic Avian Influenza A(H5N1) Virus Outbreak in New England Seals, United States. Emerg. Infect. Dis..

[114] Sevilla N., Lizarraga W., Jimenez-Vasquez V., Hurtado V., Molina I.S., Huarca L., Lope-Pari P., Vargas I., Arotinco G., Padilla-Rojas C. (2024). Highly pathogenic avian influenza A (H5N1) virus outbreak in Peru in 2022–2023. Infect. Med..

[115] Murawski A., Fabrizio T., Ossiboff R., Kackos C., Jeevan T., Jones J.C., Kandeil A., Walker D., Turner J.C.M., Patton C. (2024). Highly pathogenic avian influenza A(H5N1) virus in a common bottlenose dolphin (*Tursiops truncatus*) in Florida. Commun. Biol..

[116] Golke A., Janczak D., Szalus-Jordanow O., Dzieciatkowski T., Sapierzynski R., Moroz-Fik A., Mickiewicz M., Frymus T. (2024). Natural Infection with Highly Pathogenic Avian Influenza A/H5N1 Virus in Pet Ferrets. Viruses.

[117] Seo Y.R., Cho A.Y., Kim D.J., Si Y.J., Jeong H.S., Lee S.W., Song C.S., Lee D.H. (2025). Transmission Dynamics of Highly Pathogenic Avian Influenza A(H5N1) and A(H5N6) Viruses in Wild Birds, South Korea, 2023–2024. Emerg. Infect. Dis..

[118] Golke A., Dzieciatkowski T., Szalus-Jordanow O., Czopowicz M., Witkowski L., Zychska M., Domanska E., Janczak D., Nalbert T., Lesceu S. (2025). The Seroprevalence of Influenza A Virus Infections in Polish Cats During a Feline H5N1 Influenza Outbreak in 2023. Viruses.

[119] Brown J.D., Black A., Haman K.H., Diel D.G., Ramirez V.E., Ziejka R.S., Fenelon H.T., Rabinowitz P.M., Stevens L., Poulson R. (2024). Antibodies to Influenza A(H5N1) Virus in Hunting Dogs Retrieving Wild Fowl, Washington, USA. Emerg. Infect. Dis..

[120] Vigil K., Wu H., Aw T.G. (2024). A systematic review on global zoonotic virus-associated mortality events in marine mammals. One Health.

[121] Ly H. (2024). Highly pathogenic avian influenza H5N1 virus infections of dairy cattle and livestock handlers in the United States of America. Virulence.

[122] Nguyen T.Q., Hutter C.R., Markin A., Thomas M., Lantz K., Killian M.L., Janzen G.M., Vijendran S., Wagle S., Inderski B. (2025). Emergence and interstate spread of highly pathogenic avian influenza A(H5N1) in dairy cattle in the United States. Science.

[123] Campbell A.J., Brizuela K., Lakdawala S.S. (2025). mGem: Transmission and exposure risks of dairy cow H5N1 influenza virus. mBio.

[124] USDA Highly Pathogenic Avian Influenza Confirmed Cases in Livestock. https://www.aphis.usda.gov/livestock-poultry-disease/avian/avian-influenza/hpai-detections/hpai-confirmed-cases-livestock.

[125] Pena-Mosca F., Frye E.A., MacLachlan M.J., Rebelo A.R., de Oliveira P.S.B., Nooruzzaman M., Koscielny M.P., Zurakowski M., Lieberman Z.R., Leone W.M. (2025). The impact of highly pathogenic avian influenza H5N1 virus infection on dairy cows. Nat. Commun..

[126] Caceres C.J., Gay L.C., Faccin F.C., Regmi D., Palomares R., Perez D.R. (2024). Influenza A(H5N1) Virus Resilience in Milk after Thermal Inactivation. Emerg. Infect. Dis..

[127] Guan L., Eisfeld A.J., Pattinson D., Gu C., Biswas A., Maemura T., Trifkovic S., Babujee L., Presler R., Dahn R. (2024). Cow’s Milk Containing Avian Influenza A(H5N1) Virus—Heat Inactivation and Infectivity in Mice. N. Engl. J. Med..

[128] Le Sage V., Campbell A.J., Reed D.S., Duprex W.P., Lakdawala S.S. (2024). Persistence of Influenza H5N1 and H1N1 Viruses in Unpasteurized Milk on Milking Unit Surfaces. Emerg. Infect. Dis..

[129] Nooruzzaman M., de Oliveira P.S.B., Butt S.L., Martin N.H., Alcaine S.D., Walker S.P., Diel D.G. (2025). H5N1 influenza virus stability and transmission risk in raw milk and cheese. Nat. Med..

[130] Spackman E., Jones D.R., McCoig A.M., Colonius T.J., Goraichuk I.V., Suarez D.L. (2024). Characterization of highly pathogenic avian influenza virus in retail dairy products in the US. J. Virol..

[131] Morse J., Coyle J., Mikesell L., Stoddard B., Eckel S., Weinberg M., Kuo J., Riner D., Margulieux K., Stricklen J. (2024). Influenza A(H5N1) Virus Infection in Two Dairy Farm Workers in Michigan. N. Engl. J. Med..

[132] Uyeki T.M., Milton S., Abdul Hamid C., Reinoso Webb C., Presley S.M., Shetty V., Rollo S.N., Martinez D.L., Rai S., Gonzales E.R. (2024). Highly Pathogenic Avian Influenza A(H5N1) Virus Infection in a Dairy Farm Worker. N. Engl. J. Med..

[133] Brock N., Pulit-Penaloza J.A., Belser J.A., Pappas C., Sun X., Kieran T.J., Zeng H., De La Cruz J.A., Hatta Y., Di H. (2025). Avian Influenza A(H5N1) Isolated from Dairy Farm Worker, Michigan. Emerg. Infect. Dis..

[134] CDC H5 Bird Flu: Current Situation. https://www.cdc.gov/bird-flu/situation-summary/index.html.

[135] Di Guardo G. (2025). Highly Pathogenic Avian Influenza A(H5N1) Virus: How Far Are We from a New Pandemic?. Vet. Sci..

[136] Esaki M., Okuya K., Tokorozaki K., Haraguchi Y., Ito J., Ozawa M. (2025). Surveillance of avian influenza viruses in the Izumi plain reveals the role of wild ducks in the introduction of H5N1 HPAIVs during the 2023/24 winter season. Comp. Immunol. Microbiol. Infect. Dis..

[137] Marandino A., Tomas G., Panzera Y., Williman J., Dezordi F.Z., Wallau G.L., Rodriguez S., Perez R., Bassetti L., Negro R. (2025). Converging Transmission Routes of the Highly Pathogenic Avian Influenza H5N1 Clade 2.3.4.4b Virus in Uruguay: Phylogeographic Insights into Its Spread Across South America. Pathogens.

[138] Jindal M., Stone H., Lim S., MacIntyre C.R. (2025). A Geospatial Perspective Toward the Role of Wild Bird Migrations and Global Poultry Trade in the Spread of Highly Pathogenic Avian Influenza H5N1. Geohealth.

[139] Reperant L.A., van Amerongen G., van de Bildt M.W., Rimmelzwaan G.F., Dobson A.P., Osterhaus A.D., Kuiken T. (2008). Highly pathogenic avian influenza virus (H5N1) infection in red foxes fed infected bird carcasses. Emerg. Infect. Dis..

[140] Eisfeld A.J., Biswas A., Guan L., Gu C., Maemura T., Trifkovic S., Wang T., Babujee L., Dahn R., Halfmann P.J. (2024). Pathogenicity and transmissibility of bovine H5N1 influenza virus. Nature.

[141] Restori K.H., Septer K.M., Field C.J., Patel D.R., VanInsberghe D., Raghunathan V., Lowen A.C., Sutton T.C. (2024). Risk assessment of a highly pathogenic H5N1 influenza virus from mink. Nat. Commun..

[142] Hostyn P., Steensels M., Lambrecht B. (2025). Detection, transmission and spread of airborne avian influenza and Newcastle disease viruses: Experimental and field investigations. Vet. Res..

[143] Tosheva I.I., Filaire F., Rijnink W.F., de Meulder D., van Kekem B., Bestebroer T.M., Funk M., Spronken M.I., Caceres C.J., Perez D.R. (2025). Influenza A(H5N1) shedding in air corresponds to transmissibility in mammals. Nat. Microbiol..

[144] Yuen K.Y., Chan P.K., Peiris M., Tsang D.N., Que T.L., Shortridge K.F., Cheung P.T., To W.K., Ho E.T., Sung R. (1998). Clinical features and rapid viral diagnosis of human disease associated with avian influenza A H5N1 virus. Lancet.

[145] Bruno A., Alfaro-Nunez A., de Mora D., Armas R., Olmedo M., Garces J., Garcia-Bereguiain M.A. (2023). First case of human infection with highly pathogenic H5 avian Influenza A virus in South America: A new zoonotic pandemic threat for 2023?. J. Travel Med..

[146] Jassem A.N., Roberts A., Tyson J., Zlosnik J.E.A., Russell S.L., Caleta J.M., Eckbo E.J., Gao R., Chestley T., Grant J. (2025). Critical Illness in an Adolescent with Influenza A(H5N1) Virus Infection. N. Engl. J. Med..

[147] Rolfes M.A., Kniss K., Kirby M.K., Garg S., Reinhart K., Davis C.T., Murray E.L., Wadford D.A., Harriman K., Zhu S. (2025). Human infections with highly pathogenic avian influenza A(H5N1) viruses in the United States from March 2024 to May 2025. Nat. Med..

[148] Bamford C.C.G., Fenney N., Coleman J., Fox-Clarke C., Dickens J., Fedak M., Fretwell P., Huckstadt L., Hollyman P. (2025). Highly Pathogenic Avian Influenza Viruses (HPAIV) Associated with Major Southern Elephant Seal Decline at South Georgia. Commun. Biol..

[149] Ducatez M., Fusaro A., Gonzales J.L., Kuiken T., Stahl K., Staubach C., Terregino C., Kohnle L., European Food Safety Authority, European Union Reference Laboratory for Avian Influenza (2025). Unprecedented high level of highly pathogenic avian influenza in wild birds in Europe during the 2025 autumn migration. EFSA J..

[150] Kandeil A., Patton C., Jones J.C., Jeevan T., Harrington W.N., Trifkovic S., Seiler J.P., Fabrizio T., Woodard K., Turner J.C. (2023). Rapid evolution of A(H5N1) influenza viruses after intercontinental spread to North America. Nat. Commun..

[151] Kristensen C., Jensen H.E., Trebbien R., Webby R.J., Larsen L.E. (2024). Avian and Human Influenza A Virus Receptors in Bovine Mammary Gland. Emerg. Infect. Dis..

[152] Nelli R.K., Harm T.A., Siepker C., Groeltz-Thrush J.M., Jones B., Twu N.C., Nenninger A.S., Magstadt D.R., Burrough E.R., Pineyro P.E. (2024). Sialic Acid Receptor Specificity in Mammary Gland of Dairy Cattle Infected with Highly Pathogenic Avian Influenza A(H5N1) Virus. Emerg. Infect. Dis..

[153] Rios Carrasco M., Grone A., van den Brand J.M.A., de Vries R.P. (2024). The mammary glands of cows abundantly display receptors for circulating avian H5 viruses. J. Virol..

[154] Imai M., Ueki H., Ito M., Iwatsuki-Horimoto K., Kiso M., Biswas A., Trifkovic S., Cook N., Halfmann P.J., Neumann G. (2025). Highly pathogenic avian H5N1 influenza A virus replication in ex vivo cultures of bovine mammary gland and teat tissues. Emerg. Microbes Infect..

[155] Di Genova C., Warren C.J., Johnson S., Riccio S., Roper K., Thomas S.S., Schlachter A.L., Jorge D., Ralh K., Hassan J. (2025). Pigeons exhibit low susceptibility and poor transmission capacity for H5N1 clade 2.3.4.4b high pathogenicity avian influenza virus. J. Gen. Virol..

[156] Fabrizio T.P., Kandeil A., Harrington W.N., Jones J.C., Jeevan T., Andreev K., Seiler P., Fogo J., Davis M.L., Crumpton J.C. (2025). Genotype B3.13 influenza A(H5N1) viruses isolated from dairy cattle demonstrate high virulence in laboratory models, but retain avian virus-like properties. Nat. Commun..

[157] Goldin K., van Tol S., Johnson R.C., Mukesh R.K., Cooper K.G., Gallogly S., Schulz J.E., Prado-Smith J., Martens C., Saturday G. (2025). Enhanced neurotropism of bovine H5N1 compared to the Vietnam H5N1 isolate in C57BL/6J mice. npj Viruses.

[158] Octaviani C.P., Huang P., Bi-Hung P., Gray G.C., Tseng C.K. (2025). Superior replication, pathogenicity, and immune evasion of a Texas dairy cattle H5N1 virus compared to a historical avian isolate. Sci. Rep..

[159] Tipih T., Mariappan V., Yinda K.C., Meade-White K., Lewis M., Okumura A., McCarthy N., Altynova E., Leventhal S.S., Bushmaker T. (2025). Highly pathogenic avian influenza H5N1 clade 2.3.4.4b genotype B3.13 is highly virulent for mice, rapidly causing acute pulmonary and neurologic disease. Nat. Commun..

[160] Andersen H., Aid M., Stone J.J., Lyons C.E., Berlied A., Nkolola J., Lasrado N., Peterson M., Pessaint L., Kitajewski C. (2025). Immunopathogenesis of lethal H5N1 avian influenza virus clade 2.3.4.4b infection in macaques. Immunity.

[161] Alkie T.N., Embury-Hyatt C., Signore A.V., Ramos D., Moffat E., Raj S., Gebrebrhan H., Ayilara I., Hisanaga T., Erdelyan C.N.G. (2025). Dairy cow- and avian-origin clade 2.3.4.4b H5N1 induce severe mastitis in lactating goats and transmission to suckling goats. Cell Rep..

[162] Gu C., Maemura T., Guan L., Eisfeld A.J., Biswas A., Kiso M., Uraki R., Ito M., Trifkovic S., Wang T. (2024). A human isolate of bovine H5N1 is transmissible and lethal in animal models. Nature.

[163] Mostafa A., Barre R.S., Allue-Guardia A., Escobedo R.A., Shivanna V., Rothan H., Castro E.M., Ma Y., Cupic A., Jackson N. (2025). Replication kinetics, pathogenicity and virus-induced cellular responses of cattle-origin influenza A(H5N1) isolates from Texas, United States. Emerg. Microbes Infect..

[164] Baker A.L., Arruda B., Palmer M.V., Boggiatto P., Sarlo Davila K., Buckley A., Ciacci Zanella G., Snyder C.A., Anderson T.K., Hutter C.R. (2025). Dairy cows inoculated with highly pathogenic avian influenza virus H5N1. Nature.

[165] Halwe N.J., Cool K., Breithaupt A., Schon J., Trujillo J.D., Nooruzzaman M., Kwon T., Ahrens A.K., Britzke T., McDowell C.D. (2025). H5N1 clade 2.3.4.4b dynamics in experimentally infected calves and cows. Nature.

[166] Husain M. (2025). Influenza A Virus Non-structural 1 Protein: A Key Viral Weapon Against Host Pathways. Discov. Med..

[167] Seo S.H., Hoffmann E., Webster R.G. (2002). Lethal H5N1 influenza viruses escape host anti-viral cytokine responses. Nat. Med..

[168] Lipatov A.S., Andreansky S., Webby R.J., Hulse D.J., Rehg J.E., Krauss S., Perez D.R., Doherty P.C., Webster R.G., Sangster M.Y. (2005). Pathogenesis of Hong Kong H5N1 influenza virus NS gene reassortants in mice: The role of cytokines and B- and T-cell responses. J. Gen. Virol..

[169] Li Z., Jiang Y., Jiao P., Wang A., Zhao F., Tian G., Wang X., Yu K., Bu Z., Chen H. (2006). The NS1 gene contributes to the virulence of H5N1 avian influenza viruses. J. Virol..

[170] Twu K.Y., Kuo R.L., Marklund J., Krug R.M. (2007). The H5N1 influenza virus NS genes selected after 1998 enhance virus replication in mammalian cells. J. Virol..

[171] Jiao P., Tian G., Li Y., Deng G., Jiang Y., Liu C., Liu W., Bu Z., Kawaoka Y., Chen H. (2008). A single-amino-acid substitution in the NS1 protein changes the pathogenicity of H5N1 avian influenza viruses in mice. J. Virol..

[172] Dankar S.K., Wang S., Ping J., Forbes N.E., Keleta L., Li Y., Brown E.G. (2011). Influenza A virus NS1 gene mutations F103L and M106I increase replication and virulence. Virol. J..

[173] Spesock A., Malur M., Hossain M.J., Chen L.M., Njaa B.L., Davis C.T., Lipatov A.S., York I.A., Krug R.M., Donis R.O. (2011). The virulence of 1997 H5N1 influenza viruses in the mouse model is increased by correcting a defect in their NS1 proteins. J. Virol..

[174] Dankar S.K., Miranda E., Forbes N.E., Pelchat M., Tavassoli A., Selman M., Ping J., Jia J., Brown E.G. (2013). Influenza A/Hong Kong/156/1997(H5N1) virus NS1 gene mutations F103L and M106I both increase IFN antagonism, virulence and cytoplasmic localization but differ in binding to RIG-I and CPSF30. Virol. J..

[175] Wang B.X., Wei L., Kotra L.P., Brown E.G., Fish E.N. (2017). A Conserved Residue, Tyrosine (Y) 84, in H5N1 Influenza A Virus NS1 Regulates IFN Signaling Responses to Enhance Viral Infection. Viruses.

[176] Long J.X., Peng D.X., Liu Y.L., Wu Y.T., Liu X.F. (2008). Virulence of H5N1 avian influenza virus enhanced by a 15-nucleotide deletion in the viral nonstructural gene. Virus Genes..

[177] Zhu Q., Yang H., Chen W., Cao W., Zhong G., Jiao P., Deng G., Yu K., Yang C., Bu Z. (2008). A naturally occurring deletion in its NS gene contributes to the attenuation of an H5N1 swine influenza virus in chickens. J. Virol..

[178] Trapp S., Soubieux D., Marty H., Esnault E., Hoffmann T.W., Chandenier M., Lion A., Kut E., Quere P., Larcher T. (2014). Shortening the unstructured, interdomain region of the non-structural protein NS1 of an avian H1N1 influenza virus increases its replication and pathogenicity in chickens. J. Gen. Virol..

[179] Wang J., Zeng Y., Xu S., Yang J., Wang W., Zhong B., Ge J., Yin L., Bu Z., Shu H.B. (2018). A Naturally Occurring Deletion in the Effector Domain of H5N1 Swine Influenza Virus Nonstructural Protein 1 Regulates Viral Fitness and Host Innate Immunity. J. Virol..

[180] Chen S., Miao X., Huangfu D., Zhao X., Zhang M., Qin T., Peng D., Liu X. (2021). H5N1 avian influenza virus without 80–84 amino acid deletion at the NS1 protein hijacks the innate immune system of dendritic cells for an enhanced mammalian pathogenicity. Transbound. Emerg. Dis..

[181] Husain M. (2020). Host factors involved in influenza virus infection. Emerg. Top. Life Sci..

[182] Heindl M.R., Bottcher-Friebertshauser E. (2023). The role of influenza-A virus and coronavirus viral glycoprotein cleavage in host adaptation. Curr. Opin. Virol..

[183] Wood G.W., McCauley J.W., Bashiruddin J.B., Alexander D.J. (1993). Deduced amino acid sequences at the haemagglutinin cleavage site of avian influenza A viruses of H5 and H7 subtypes. Arch. Virol..

[184] Senne D.A., Panigrahy B., Kawaoka Y., Pearson J.E., Suss J., Lipkind M., Kida H., Webster R.G. (1996). Survey of the hemagglutinin (HA) cleavage site sequence of H5 and H7 avian influenza viruses: Amino acid sequence at the HA cleavage site as a marker of pathogenicity potential. Avian Dis..

[185] Hatta M., Gao P., Halfmann P., Kawaoka Y. (2001). Molecular basis for high virulence of Hong Kong H5N1 influenza A viruses. Science.

[186] Suarez D.L., Perdue M.L., Cox N., Rowe T., Bender C., Huang J., Swayne D.E. (1998). Comparisons of highly virulent H5N1 influenza A viruses isolated from humans and chickens from Hong Kong. J. Virol..

[187] Torchetti M.K., Killian M.L., Dusek R.J., Pedersen J.C., Hines N., Bodenstein B., White C.L., Ip H.S. (2015). Novel H5 Clade 2.3.4.4 Reassortant (H5N1) Virus from a Green-Winged Teal in Washington, USA. Genome Announc..

[188] Izumi H., Nafie L.A., Dukor R.K. (2025). Conformational Variability Prediction of H5N1 Avian Influenza A Virus Hemagglutinins with Amino Acid Mutations Using SSSCPreds. ACS Omega.

[189] Subbarao E.K., London W., Murphy B.R. (1993). A single amino acid in the PB2 gene of influenza A virus is a determinant of host range. J. Virol..

[190] Kim D.H., Lee D.Y., Seo Y., Song C.S., Lee D.H. (2025). Immediate PB2-E627K amino acid substitution after single infection of highly pathogenic avian influenza H5N1 clade 2.3.4.4b in mice. Virol. J..

[191] Shichinohe S., Hiono T., Itoh Y., Takada K., Kida Y., Wang P., Motooka D., Isoda N., Takada A., Sakoda Y. (2025). Characterization of H5N1 high pathogenicity avian influenza virus belonging to clade 2.3.4.4b isolated from Ezo red fox in Japan in a mouse model. Microbiol. Spectr..

[192] Hu X., Saxena A., Magstadt D.R., Gauger P.C., Burrough E.R., Zhang J., Siepker C., Mainenti M., Gorden P.J., Plummer P.J. (2024). Genomic characterization of highly pathogenic avian influenza A H5N1 virus newly emerged in dairy cattle. Emerg. Microbes Infect..

[193] Bayoumi M., Barre R.S., Escobedo R.A., Shivanna V., Jackson N., Ye C., Garcia-Sastre A., Mostafa A., Martinez-Sobrido L. (2025). Identification of amino acid residues in polymerase PB2 responsible for differential replication and pathogenicity of avian influenza virus H5N1 isolated from human and cattle in Texas, US. Emerg. Microbes Infect..

[194] Kim Y.I., Jang S.G., Kwon W., Kim J., Park D., Choi I., Choi J.H., Gil J., Yu M., Jeong B. (2025). PB2 and NP of North American H5N1 virus drive immune cell replication and systemic infections. Sci. Adv..

[195] Song H., Hao T., Han P., Wang H., Zhang X., Li X., Wang Y., Chen J., Li Y., Jin X. (2025). Receptor binding, structure, and tissue tropism of cattle-infecting H5N1 avian influenza virus hemagglutinin. Cell.

[196] Banyard A.C., Coombes H., Terrey J., McGinn N., Seekings J., Clifton B., Mollett B.C., Genova C.D., Sainz-Dominguez P., Worsley L. (2025). Detection of clade 2.3.4.4b H5N1 high pathogenicity avian influenza virus in a sheep in Great Britain, 2025. Emerg. Microbes Infect..

[197] Good M.R., Fernandez-Quintero M.L., Ji W., Rodriguez A.J., Han J., Ward A.B., Guthmiller J.J. (2024). A single mutation in dairy cow-associated H5N1 viruses increases receptor binding breadth. Nat. Commun..

[198] Li Z., Chen H., Jiao P., Deng G., Tian G., Li Y., Hoffmann E., Webster R.G., Matsuoka Y., Yu K. (2005). Molecular basis of replication of duck H5N1 influenza viruses in a mammalian mouse model. J. Virol..

[199] Thompson A.J., Paulson J.C. (2021). Adaptation of influenza viruses to human airway receptors. J. Biol. Chem..

[200] Rogers G.N., Paulson J.C., Daniels R.S., Skehel J.J., Wilson I.A., Wiley D.C. (1983). Single amino acid substitutions in influenza haemagglutinin change receptor binding specificity. Nature.

[201] Shinya K., Hatta M., Yamada S., Takada A., Watanabe S., Halfmann P., Horimoto T., Neumann G., Kim J.H., Lim W. (2005). Characterization of a human H5N1 influenza A virus isolated in 2003. J. Virol..

[202] Stevens J., Blixt O., Tumpey T.M., Taubenberger J.K., Paulson J.C., Wilson I.A. (2006). Structure and receptor specificity of the hemagglutinin from an H5N1 influenza virus. Science.

[203] Yamada S., Suzuki Y., Suzuki T., Le M.Q., Nidom C.A., Sakai-Tagawa Y., Muramoto Y., Ito M., Kiso M., Horimoto T. (2006). Haemagglutinin mutations responsible for the binding of H5N1 influenza A viruses to human-type receptors. Nature.

[204] Bruno A., de Mora D., Garcia-Bereguiain M.A., Cristina J. (2025). Phylogenetic and Mutation Analysis of Hemagglutinin Gene from Highly Pathogenic Avian Influenza Virus H5 Clade 2.3.4.4b in South America. Viruses.

[205] Wang L., Hatta M., Feng C., Carney P., Almanzar-Jordan M.R., Wang J., Hossain J., Atteberry G., Assad-Garcia N., Sheffield S. (2025). Impact of naturally occurring hemagglutinin substitutions on antigenicity and fitness of influenza A(H5N1) virus. npj Viruses.

[206] Pulit-Penaloza J.A., Belser J.A., Brock N., Kieran T.J., Sun X., Pappas C., Zeng H., Carney P., Chang J., Bradley-Ferrell B. (2024). Transmission of a human isolate of clade 2.3.4.4b A(H5N1) virus in ferrets. Nature.

[207] Zhu B., Fung K., Feng H.H., Beatty J.A., Hill F., Tse A.C.N., Brackman C.J., Sit T.H.C., Poujade A., Gaide N. (2025). The hemagglutinin proteins of clades 1 and 2.3.4.4b H5N1 highly pathogenic avian influenza viruses exhibit comparable attachment patterns to avian and mammalian tissues. J. Virol..

[208] Yang J., Qureshi M., Kolli R., Peacock T.P., Sadeyen J.R., Carter T., Richardson S., Daines R., Barclay W.S., Brown I.H. (2025). The haemagglutinin gene of bovine-origin H5N1 influenza viruses currently retains receptor-binding and pH-fusion characteristics of avian host phenotype. Emerg. Microbes Infect..

[209] Santos J.J.S., Wang S., McBride R., Adams L., Harvey R., Zhao Y., Wrobel A.G., Gamblin S., Skehel J., Lewis N.S. (2025). Bovine H5N1 binds poorly to human-type sialic acid receptors. Nature.

[210] Chopra P., Ray S.D., Page C.K., Shepard J.D., Kandeil A., Jeevan T., Bowman A.S., Ellebedy A.H., Webby R.J., de Vries R.P. (2025). Receptor-binding specificity of a bovine influenza A virus. Nature.

[211] Andreev K., Jones J.C., Seiler P., Kandeil A., Turner J.C.M., Barman S., Rubrum A.M., Webby R.J., Govorkova E.A. (2024). Antiviral Susceptibility of Highly Pathogenic Avian Influenza A(H5N1) Viruses Circulating Globally in 2022–2023. J. Infect. Dis..

[212] Jones J.C., Andreev K., Fabrizio T.P., Bowman A.S., Govorkova E.A., Webby R.J. (2025). Baloxavir improves disease outcomes in mice after intranasal or ocular infection with Influenza A virus H5N1-contaminated cow’s milk. Nat. Microbiol..

[213] Qiu Y.Z., Yin W.D. (2008). Safety and immunogenicity of Sinovac’s prototype pandemic influenza H5N1 vaccines: A review on clinical trials. Influenza Other Respir. Viruses.

[214] Barron M. Avian Influenza (H5N1) Vaccines: What’s the Status? 2025. https://asm.org/articles/2025/march/avian-influenza-h5n1-vaccines-what-status.

[215] Nunn A., Sinilaite A., Warshawsky B., Salvadori M.I., Bogoch I.I., Siu W. (2025). Avian influenza and use of the H5N1 vaccine to prevent zoonotic infection in Canada. Can. Med. Assoc. J..

[216] Khurana S., King L.R., Manischewitz J., Posadas O., Mishra A.K., Liu D., Beigel J.H., Rappuoli R., Tsang J.S., Golding H. (2024). Licensed H5N1 vaccines generate cross-neutralizing antibodies against highly pathogenic H5N1 clade 2.3.4.4b influenza virus. Nat. Med..

[217] Huang X., Yu D., Pan L., Wu X., Li J., Wang D., Liu L., Zhao C., Huang W. (2025). Increase in H5N1 vaccine antibodies confers cross-neutralization of highly pathogenic avian influenza H5N1. Nat. Commun..

[218] Hatta M., Hatta Y., Choi A., Hossain J., Feng C., Keller M.W., Ritter J.M., Huang Y., Fang E., Pusch E.A. (2024). An influenza mRNA vaccine protects ferrets from lethal infection with highly pathogenic avian influenza A(H5N1) virus. Sci. Transl. Med..

[219] Lasrado N., Wang L., Liu J., Rossler A., Chaudhari J., Wang Q., Stone J.J., Granados-Contreras F.A., Wu J., Cabrera-Barragan D.N. (2025). An intramuscular prime and mucosal boost vaccine regimen protects against lethal clade 2.3.4.4b H5N1 challenge in cynomolgus macaques. Sci. Transl. Med..

[220] Mostafa A., Ye C., Barre R.S., Shivanna V., Meredith R., Platt R.N., Escobedo R.A., Bayoumi M., Castro E.M., Jackson N. (2025). A live attenuated NS1-deficient vaccine candidate for cattle-origin influenza A (H5N1) clade 2.3.4.4.b viruses. npj Vaccines.

[221] Liu Y., Deng S., Ren S., Tam R.C., Liu S., Zhang A.J., To K.K., Yuen K.Y., Chen H., Wang P. (2025). Intranasal influenza virus-vectored vaccine offers protection against clade 2.3.4.4b H5N1 infection in small animal models. Nat. Commun..

[222] Deming M.E., Toapanta F.R., Pasetti M., Golding H., Khurana S., Hamouda T., Fattom A., Liang Y., Tennant S.M., McGilvray M.F. (2025). An intranasal adjuvanted, recombinant influenza A/H5 vaccine primes against diverse H5N1 clades: A phase I trial. Nat. Commun..

[223] Kim C.U., Choi W.S., Lee P., Jeong J.H., Seo Y.B., Choi Y.W., Sohn M.H., Choi S.Y., Jeong A.Y., Song M.S. (2025). A consensus HA DNA vaccine targeting clade 2.3.4.4 H5Nx influenza viruses provides broad cross-clade protection in mice and ferrets. Biomed. Pharmacother..

[224] Rudometov A.P., Litvinova V.R., Gudymo A.S., Ivanova K.I., Rudometova N.B., Kisakov D.N., Borgoyakova M.B., Kisakova L.A., Yakovlev V.A., Tigeeva E.V. (2025). Dose-Dependent Effect of DNA Vaccine pVAX-H5 Encoding a Modified Hemagglutinin of Influenza A (H5N8) and Its Cross-Reactivity Against A (H5N1) Influenza Viruses of Clade 2.3.4.4b. Viruses.

[225] Mahmoud S.H., Khalil A.A., Abo Shama N.M., El Sayed M.F., Soliman R.A., Hagag N.M., Yehia N., Naguib M.M., Arafa A.S., Ali M.A. (2023). Immunogenicity and Cross-Protective Efficacy Induced by an Inactivated Recombinant Avian Influenza A/H5N1 (Clade 2.3.4.4b) Vaccine against Co-Circulating Influenza A/H5Nx Viruses. Vaccines.

[226] Ahmadivand S., Fux R., Palic D. (2024). Ferritin Vaccine Platform for Animal and Zoonotic Viruses. Vaccines.

[227] Paran N., Wirblich C., Olal C., Tarquinio A., Lohmeyer K.H., Kurup D., Schultz-Cherry S., Shittu I., Gray G.C., Bente D.A. (2025). Immunogenicity and safety of a rabies-based highly pathogenic influenza A virus H5 vaccine in cattle. npj Vaccines.

[228] Valentin J., Ingrao F., Rauw F., Lambrecht B. (2024). Protection conferred by an H5 DNA vaccine against highly pathogenic avian influenza in chickens: The effect of vaccination schedules. Vaccine.

[229] Lee J., Lee C.W., Lee S., Ibrahim S., Suarez D.L., Spackman E. (2025). The Efficacy of Inactivated Vaccine Against H5 Clade 2.3.4.4b Highly Pathogenic Avian Influenza Virus in Turkeys. Avian Dis..

[230] Piesche R., Cazaban C., Frizzo da Silva L., Ramirez-Martinez L., Hufen H., Beer M., Harder T., Grund C. (2025). Immunogenicity and Protective Efficacy of Five Vaccines Against Highly Pathogenic Avian Influenza Virus H5N1, Clade 2.3.4.4b, in Fattening Geese. Vaccines.

[231] Elfeil W.M., Safwat M., Sedeek A., Adel A., Hassan H., Zain El-Abideen M.A., Ali I., Hisham I., Arafa A., Selim A. (2025). Efficacy of a multi-clade inactivated recombinant vaccine against the circulating highly pathogenic influenza A/H5N1 of clade 2.3.4.4b in poultry. Avian Pathol..

[232] Brice D.C., Andreev K., Miller L., Patton C., Seiler P., Garcia T., LeBlanc M.L., Hibler T., Ozdemir E., Mandarano A.H. (2025). Immunogenicity and efficacy of commercial poultry avian influenza vaccines against HPAI A(H5N1) clade 2.3.4.4b viruses in Mexico. Virology.

[233] Abu-Shmais A.A., Freeman G., Creanga A., Vukovich M.J., Malla T., Mantus G.E., Shimberg G.D., Gillespie R.A., Guerra Canedo V., Dadonaite B. (2025). Cross-neutralizing and potent human monoclonal antibodies against historical and emerging H5Nx influenza viruses. Nat. Microbiol..

[234] Kojima N., Blumberg A., Radcliffe R., Flannery B., Uyeki T.M. (2024). US Public Health Preparedness and Response to Highly Pathogenic Avian Influenza A(H5N1) Viruses. JAMA.

[235] Dhillon R.S., Karan A., Garry R.F., Srikrishna D. (2025). Steps to prevent and respond to an H5N1 epidemic in the USA. Nat. Med..

[236] Ramey A.M., Prosser D.J., Hubbard L.E., Vazquez-Meves G., George A., Hopkins M.C. (2025). U.S. Geological Survey science strategy to address highly pathogenic avian influenza and its effects on wildlife health. Geol. Surv. Circ..

[237] Park S.D., Oh Y., Yoo D.S. (2025). Combating Highly Pathogenic Avian Influenza in South Korea: A 15-year retrospective and forward-looking study. Prev. Vet. Med..

[238] Huang P., Sun L., Li J., Wu Q., Rezaei N., Jiang S., Pan C. (2023). Potential cross-species transmission of highly pathogenic avian influenza H5 subtype (HPAI H5) viruses to humans calls for the development of H5-specific and universal influenza vaccines. Cell Discov..

[239] Li C., Bu Z., Chen H. (2014). Avian influenza vaccines against H5N1 ‘bird flu’. Trends Biotechnol..

[240] Zeng X.-Y., Chen X.-H., Ma S.-J., Wu J.-J., Bao H.-M., Pan S.-X., Liu Y.-J., Deng G.-H., Shi J.-Z., Chen P.-C. (2020). Protective efficacy of an H5/H7 trivalent inactivated vaccine produced from Re-11, Re-12, and H7-Re2 strains against challenge with different H5 and H7 viruses in chickens. J. Integr. Agric..

[241] Badillo B.A.M., García D.L.H., García R.A.M., Pineda G.O., Ramiro C.J.A., Castillo J.C., Hernández M.S., López R.N., López A.G. (2025). H5N1 highly pathogenic avian influenza vaccination: Seroresponse of mexican poultry in the 2022–2024. Vaccine X.

[242] Marschik T., Sawodny S., Kopacka I., Hoflechner-Poltl A., Revilla-Fernandez S., Zimpernik I., Schmoll F., Kasbohrer A. (2025). Cost assessment of a preventive vaccination program against highly pathogenic avian influenza in Austrian poultry farms. Prev. Vet. Med..

[243] Nielsen S.S., Alvarez J., Bicout D.J., Calistri P., Canali E., Drewe J.A., Garin-Bastuji B., Gonzales Rojas J.L., Gortázar C., EFSA Panel on Animal Health and Animal Welfare (AHAW), European Union Reference Laboratory for Avian Influenza (2023). Vaccination of poultry against highly pathogenic avian influenza—Part 1. Available vaccines and vaccination strategies. EFSA J..

[244] Lin T.H., Zhu X., Wang S., Zhang D., McBride R., Yu W., Babarinde S., Paulson J.C., Wilson I.A. (2024). A single mutation in bovine influenza H5N1 hemagglutinin switches specificity to human receptors. Science.

[245] Rios Carrasco M., Lin T.H., Zhu X., Gabarroca Garcia A., Uslu E., Liang R., Spruit C.M., Richard M., Boons G.J., Wilson I.A. (2025). The Q226L mutation can convert a highly pathogenic H5 2.3.4.4e virus to bind human-type receptors. Proc. Natl. Acad. Sci. USA.

[246] Vanstreels R.E.T., Nelson M.I., Artuso M.C., Marchione V.D., Piccini L.E., Benedetti E., Crespo-Bellido A., Pierdomenico A., Wolff T., Uhart M.M. (2025). Novel Highly Pathogenic Avian Influenza A(H5N1) Virus, Argentina, 2025. Emerg. Infect. Dis..

[247] Rivetti A.V., Reischak D., Carnegie L., Otaka J.N.P., Domingues C.S., Cardoso F.G., da Silva A.L.S., Camillo S.C.A., Goes-Neto A., Camargos M.F. (2025). Genomic diversity and reassortment of highly pathogenic avian influenza A/H5N1 virus (clade 2.3.4.4b) in Brazil: Evidence of multiple introductions and intra-epidemic reassortment in 2025. Virology.

[248] Li Z.N., Liu F., Jung Y.J., Jefferson S., Holiday C., Gross F.L., Tzeng W.P., Carney P., Kates A., York I.A. (2025). Pre-existing cross-reactive immunity to highly pathogenic avian influenza 2.3.4.4b A(H5N1) virus in the United States. Nat. Commun..

[249] Restori K.H., Weaver V., Patel D.R., Merrbach G.A., Septer K.M., Field C.J., Bernabe M.J., Kronthal E.M., Minns A., Lindner S.E. (2025). Preexisting immunity to the 2009 pandemic H1N1 virus reduces susceptibility to H5N1 infection and disease in ferrets. Sci. Transl. Med..

[250] Fosse J.H., Romo G., Bonfante F., Myhrvold I.K., Soetart K.S., Udjus K., Tonnessen R. (2025). Detection of Antibodies Specific to H5 Avian Influenza Virus in a Sheep in Norway, June 2024, 11 Months After an Outbreak of Highly Pathogenic Avian Influenza in a Nearby Seabird Colony. Influenza Other Respir. Viruses.

[251] Graaf A., Piesche R., Sehl-Ewert J., Grund C., Pohlmann A., Beer M., Harder T. (2023). Low Susceptibility of Pigs against Experimental Infection with HPAI Virus H5N1 Clade 2.3.4.4b. Emerg. Infect. Dis..

[252] Kwon T., Trujillo J.D., Carossino M., Lyoo E.L., McDowell C.D., Cool K., Matias-Ferreyra F.S., Jeevan T., Morozov I., Gaudreault N.N. (2024). Pigs are highly susceptible to but do not transmit mink-derived highly pathogenic avian influenza virus H5N1 clade 2.3.4.4b. Emerg. Microbes Infect..

[253] Kwon T., Trujillo J.D., Carossino M., Machkovech H.M., Cool K., Lyoo E.L., Singh G., Kafle S., Elango S., Vediyappan G. (2025). Pathogenicity and transmissibility of bovine-derived HPAI H5N1 B3.13 virus in pigs. Emerg. Microbes Infect..

[254] Seeger R.M., Hagerman A.D., Johnson K.K., Pendell D.L., Marsh T.L. (2021). When poultry take a sick leave: Response costs for the 2014–2015 highly pathogenic avian influenza epidemic in the USA. Food Policy.

[255] Bellido-Martin B., Rijnink W.F., Iervolino M., Kuiken T., Richard M., Fouchier R.A.M. (2025). Evolution, spread and impact of highly pathogenic H5 avian influenza A viruses. Nat. Rev. Microbiol..

[256] Cui P., Shi J., Wang C., Zhang Y., Xing X., Kong H., Yan C., Zeng X., Liu L., Tian G. (2022). Global dissemination of H5N1 influenza viruses bearing the clade 2.3.4.4b HA gene and biologic analysis of the ones detected in China. Emerg. Microbes Infect..

[257] Ekouo J., Tague C., Yokolo H., Makeda D., Akilimali A. (2025). Effectiveness of non-pharmaceutical control strategies in highly pathogenic avian influenza (H5N1, H5N8) outbreaks. Clin. Infect. Pract..

[258] Yamaguchi E., Hayama Y., Kondo S., Yamamoto T. (2025). Risk factors for highly pathogenic avian influenza outbreaks in Japan during 2022–2023 season identified by additive Bayesian network modeling. Sci. Rep..

[259] Guinat C., Valenzuela Agui C., Briand F.X., Chakraborty D., Fourtune L., Lambert S., Jimenez Pellicer A., Rautureau S., Gerbier G., du Plessis L. (2025). Poultry farm density and proximity drive highly pathogenic avian influenza spread. Commun. Biol..

[260] Kondo S., Yamaguchi E., Hayama Y., Yamamoto T. (2025). Risk Factors for Introduction of H5N1 Highly Pathogenic Avian Influenza Virus in Japanese Commercial Layer Farms During the 2022–2023 Epidemic: A Case-Control Study. Transbound. Emerg. Dis..

[261] Gonnerman M., Mullinax J.M., Fox A., Patyk K.A., Fields V.L., McCool M.J., Torchetti M.K., Lantz K., Sullivan J.D., Prosser D.J. (2025). Avian influenza spillover into poultry: Environmental influences and biosecurity protections. One Health.

[262] Tisza M.J., Hanson B.M., Clark J.R., Wang L., Payne K., Ross M.C., Mena K.D., Gitter A., Javornik Cregeen S.J., Cormier J. (2024). Sequencing-Based Detection of Avian Influenza A(H5N1) Virus in Wastewater in Ten Cities. N. Engl. J. Med..

[263] Dhillon R.S., Karan A., Srikrishna D. (2024). Enhancing wastewater testing for H5N1 surveillance. Lancet Infect. Dis..

[264] Yeh K.B., Bahnfleth W.P., Bradford E., Cardona C., Coleman K.K., Hudson P.J., Dadonaite B., Hutchins R.J., Maresso A., MacIntyre C.R. (2025). Three things we can do now to reduce the risk of avian influenza spillovers. Proc. Natl. Acad. Sci. USA.

[265] Alfonsi T., Bernasconi A., Chiara M., Ceri S. (2025). Lightweight multiscale early warning system for influenza A spillovers. Sci. Adv..

[266] Goodman K.E., Shams S.M., Magder L.S., Baghdadi J.D., Morgan D.J., Harris A.D. (2025). Generative Artificial Intelligence-based Surveillance for Avian Influenza Across a Statewide Healthcare System. Clin. Infect. Dis..

[267] Ren W.J., Rose J.B. (2025). Call for Expanding Environmental Surveillance of H5N1: The Role of Microbial Source Tracking. Environ. Sci. Technol. Lett..

